# The evolution of vaccine strategies for colorectal cancer: from conventional approaches to the mRNA era

**DOI:** 10.3389/fimmu.2026.1778522

**Published:** 2026-05-08

**Authors:** Tengfei Wang, Yuzhou Mei, Songhao Liu, Haoyu Wang, Min Chen, Zhenguo Han

**Affiliations:** 1 Third Hospital of Shanxi Medical University, Shanxi Bethune Hospital, Shanxi Academy of Medical Sciences, Tongji Shanxi Hospital, Taiyuan, China; 2 Department of Colorectal and Anal Surgery, Shanxi Bethune Hospital, Third Hospital of Shanxi Medical University, Shanxi Academy of Medical Sciences, Taiyuan, Shanxi, China

**Keywords:** colorectal cancer, immunotherapy, mRNA vaccine, neoantigen, vaccine

## Abstract

Colorectal cancer (CRC) remains one of the leading causes of cancer-related mortality worldwide, with current treatment modalities frequently limited by tumor heterogeneity and immune evasion. Vaccine-based immunotherapy, particularly that utilizing messenger RNA (mRNA) technology, represents a promising innovative strategy due to its rapid development cycle, generally manageable safety profile in the short term, and potential for personalization. This review examines the limitations of traditional cancer vaccine platforms and contrasts them with the advantages offered by mRNA-based vaccines. It expounds on the various mechanisms through which mRNA vaccines target CRC, such as encoding tumor-associated antigens or tumor-specific neoantigens, delivering immune-stimulating cytokines, and modulating the immunosuppressive tumor microenvironment. An overview of recent clinical trials in the field of mRNA immunotherapy for colorectal cancer is provided. Preliminary evidence from early clinical studies indicates that these approaches exhibit a manageable safety profile and show signs of antitumor activity, showcasing significant potential for clinical application. Furthermore, we also discuss the challenges and prospects that mRNA vaccines are currently facing.

## 1 Introduction

Colorectal cancer (CRC) is the third most prevalent type of cancer worldwide (1). Despite significant advancements in endoscopic techniques for screening and early detection, which have contributed to a reduction in incidence in some regions, CRC remains the second leading cause of cancer-related mortality globally. Furthermore, in many developing countries, the incidence, prevalence, and mortality rates associated with CRC continue to be alarmingly high and are projected to increase further over the next decade, particularly in regions undergoing rapid economic transition (2).

The primary treatment for CRC involves surgical resection, which may be accompanied by adjuvant therapies such as chemotherapy or radiotherapy (3). Although immunotherapy combined with chemotherapy has been shown to significantly extend progression-free survival and overall survival in a subset of patients with CRC (e.g., those with deficient mismatch repair/microsatellite instability-high tumors), challenges including tumor heterogeneity and drug resistance continue to pose substantial obstacles to effective treatment for the majority of patients (4). Consequently, addressing these therapeutic challenges in individuals with advanced CRC may require alternative therapeutic strategies or new immunotherapeutic approaches (5, 6).

Vaccines traditionally function by stimulating the immune system to recognize and combat specific pathogens. Since the 18th century, vaccination has culminated in the complete eradication of smallpox and has significantly advanced the control of various infectious diseases (7). This success has inspired efforts to apply similar immunological principles to oncology, positioning vaccines as a promising frontier in cancer research. While early efforts focused on preventing virus-associated cancers, the broader goal of treating existing malignancies has driven the development of therapeutic cancer vaccines—designed to activate the immune system against established tumors. In 2010, FDA approval of Sipuleucel-T (Provenge)—the first therapeutic cancer vaccine—spurred research into neoantigen and vector-based platforms to overcome immune suppression and broaden efficacy (8). Subsequently, various platforms for cancer vaccines have been explored, each characterized by distinct mechanisms and challenges. These include cell-based vaccines, which utilize whole tumor cells or dendritic cells to present a broad array of antigens; microorganism-based vaccines, employing attenuated bacteria or viral vectors to deliver tumor antigens and provoke robust innate immune responses; exosome-based vaccines, which leverage exosomes for delivery to facilitate antigen presentation and immune activation; protein and peptide based vaccines, valued for their straightforward design, safety profile, and ease of production; and DNA-based vaccines, offering high stability and the capacity to induce sustained antigen expression (9). While each platform offers unique advantages, they are often constrained by limitations in immunogenicity, manufacturing complexity, or scalability, underscoring the imperative for more versatile and potent alternatives.

During the COVID-19 pandemic, messenger RNA (mRNA) vaccines rapidly gained prominence due to their unique advantages, including a favorable short-term safety profile, short development cycles, and flexible production capabilities (10). This attention has accelerated their application in cancer research. As a frontier technology derived from molecular biology and immunology (11), mRNA was first successfully demonstrated in an animal model using *in vitro* transcription (IVT) in 1990 (12). Over the past few decades, continuous advancements in nucleotide modification techniques and delivery strategies have significantly enhanced the efficacy, safety, and scalability of mRNA vaccines (13, 14). Collectively, these advancements underscore the growing promise of mRNA vaccines for applications in both tumor prevention and treatment.

The capacity of mRNA vaccines to engage both innate and adaptive immunity positions them as a uniquely adaptable platform. This inherent versatility, combined with their rapid and flexible manufacturing, makes them particularly attractive for personalised cancer immunotherapy. For instance, mRNA constructs encoding tumor-specific antigens (TSAs) offer a theoretical advantage: each patient translates the mRNA into proteins, which are then processed and presented in a manner that is unique and personalized. Antigen-presenting cells (APCs) can take up, process, and present these TSAs in the context of various human leukocyte antigen (HLA) class I and II molecules. If the resulting epitopes bind with high affinity to the patient’s HLA molecules, they can be recognized by T cells, leading to a broader and more efficient T-cell response *in vivo (*15–17).

In light of the profound heterogeneity and immune evasion that characterize CRC, mRNA vaccines offer a promising solution to current therapeutic challenges. Their rapid production and inherent customizability enable the development of personalized treatment platforms, facilitating the design of multi-epitope vaccines capable of targeting tumor clonal diversity (15, 18). This strategy may help reduce the risk of immune escape driven by heterogeneous antigen expression. Moreover, mRNA vaccines can activate both innate and adaptive immune pathways, eliciting robust CD8^+^ and CD4^+^ T cell responses specific to target antigens. This immune activation can promote the conversion of immunologically “cold” tumors into “hot” tumor microenvironments characterized by T cell infiltration, potentially overcoming mechanisms of immune evasion (19). Preclinical studies and early clinical trials have demonstrated promising results, underscoring their potential to activate tumor-specific immune responses and improve patient prognosis (5, 20, 21). Nonetheless, there remain challenges in augmenting immunological effects, refining delivery mechanisms and guaranteeing stability.

This review summarizes the advantages and disadvantages of different tumor vaccines, with particular emphasis on the potential biological functions underlying mRNA vaccines in CRC. We also discuss recent advancements in their clinical applications along with the challenges encountered and future directions for development in this field.

## 2 CRC vaccine strategy: from conventional to mRNA platforms

### 2.1 Conventional cancer vaccines: exploration and limitations

A wide variety of conventional vaccines for colorectal cancer exist, which can be categorized into several major types based on the final form that enters the human body: cell-based, microorganism-based, exosome-based, protein/peptide-based, and DNA-based vaccines. This section provides an overview of these conventional vaccine types and focuses on the limitations associated with each method in terms of formulation or delivery.

#### 2.1.1 Cell-based vaccines

Cell-based vaccines leverage whole cells as either the source of antigens or as the delivery vehicle. Currently developed cell-based vaccines for CRC can be broadly categorised into two types based on their formulation and delivery strategy. The first type, tumour cell vaccines, utilize whole tumour cells (often autologous or allogeneic) as the antigenic formulation (22). The second type, dendritic cell (DC) vaccines, utilize DCs as a delivery system. By loading peptides or mRNA onto dendritic cells and reinfusing them into the patient’s body, they can exert their effects (23). Of these two types, vaccines that utilize tumor cells—especially autologous tumor cells—are well-suited for personalized treatment approaches, as they can be directly matched to the patient’s specific antigenic profile (22). In various preclinical cancer models and clinical trials evaluating similar vaccines in patients with CRC, it has been observed that the anti-tumor response correlates with the immune response elicited by the vaccine (24–27). GVAX^®^ and Vigil™ serve as representative examples of this category. In a Phase I clinical trial (NCT00656123), GVAX^®^ combined with cyclophosphamide was associated with extended survival in patients following radical resection of liver metastases from colorectal cancer; however, its efficacy may be limited in patients presenting with a significant burden of metastatic disease (27). Additionally, results from two other GVAX^®^ vaccine trials indicated limited clinical efficacy, as seen in NCT02981524, NCT01966289. Vigil™, while exhibiting promising outcomes in other malignancies (NCT01309230), was terminated in CRC (28). The clinical trial registry (NCT01505166) cites a “Business Decision to pursue other indications” as the official reason^1^. This decision likely reflects the inherent and widely reported challenges within the field of autologous tumor cell vaccines, particularly their intricate manufacturing processes and the logistical hurdles associated with obtaining sufficient, viable tumor tissue from colorectal cancer patients (29).

DC vaccines leverage the capabilities of dendritic cells, which are recognized as the most potent professional APCs (30). Typically, DCs are utilized to load antigens and subsequently infuse them back into patients (31–34). In a Phase I clinical trial (NCT00558051), nine patients with invasive and recurrent malignancies received intranodal injections of DC vaccines loaded with killed autologous tumor cells, keyhole limpet hemocyanin, and pan DR helper T cell epitope. The overall cohort survival period was > 28 months (± 25 months). Among them, one patient achieved long-term disease-free survival for over 90 months post-treatment (31). In another Phase I/II clinical trial (NCT00228189), ten patients with CRC liver metastases received intradermal and intravenous injections of dendritic cells pulsed with carcinoembryonic antigen (CEA) peptide before undergoing resection of liver metastases. A significant number of CEA-specific T cells were detected in delayed-type hypersensitivity (DTH) biopsies from seven patients post-treatment, which produced substantial amounts of interferon-gamma (IFN-γ) upon stimulation with target cells loaded with CEA. Interestingly, within this study, a comparative analysis revealed that DCs transfected with CEA mRNA were not superior to CEA peptide-pulsed DCs in inducing tumor-specific immune responses (35). The underlying mechanisms for this unexpected observation remain elusive; however, they may stem from differential efficiencies in MHC class II antigen processing or the induction of distinct T-cell subsets by each vaccination strategy, as suggested by studies in other tumor types (36). These findings underscore the potential application value of DC vaccines in CRC; however, the development process for these vaccines is lengthy and necessitates autologous cell preparations, which may not align with the economic requirements for precision treatment (37).

It is noteworthy that a bioengineered allogeneic immune cell vaccine, AlloStim, has also been utilized in the treatment of colorectal cancer (NCT02380443, NCT01065441, NCT00861107)^2^. This vaccine employs allogeneic activated CD4^+^ Th1-like cells and is intentionally mismatched with the recipient to induce reactivation of the immune response through a graft-versus-host-disease-like reaction (38). Specifically, a case report associated with a Phase IIb trial documented a rare objective response to ICIs in a single patient with pMMR/MSS mCRC following AlloStim^®^ treatment (39). While this individual case suggests a potential for therapeutic sensitization, preliminary results from the NCT02380443 trial, as reported on ClinicalTrials.gov, indicate that a majority of participants experienced varying degrees of adverse reactions^3^. These observations highlight the necessity for larger, controlled clinical trials to rigorously establish both the safety profile and the therapeutic efficacy of this combination strategy.

#### 2.1.2 Microorganism-based vaccines

Microorganism CRC vaccines include bacterial, viral, and yeast-based vaccines. These vaccines possess inherent immunogenicity, and their genetic material can be modified to incorporate cancer antigens (40). The development of viral vector vaccines has progressed relatively rapidly (41, 42). A Phase I clinical trial involving patients with CRC demonstrated that a recombinant avian pox virus is safe but elicits only limited T-cell responses in cancer patients (42). This observation may be partly attributed to the induction of neutralizing antiviral antibodies—a known challenge for viral vector platforms. Yeast-based vaccines have been shown to be safe in multiple Phase I clinical trials (43–45). An ongoing Phase I clinical trial aims to integrate the yeast vaccine with personalized treatment approaches (NCT03552718). Concurrently, recent preclinical research indicates that antigen-anchored yeast vaccines can significantly activate intestinal DCs, thereby amplifying the immune response (46). Bacteria-based vaccines, such as *Salmonella typhimurium*, have been utilized in other malignancies due to their ability to achieve tumor-specific colonization (47, 48). However, microorganism-based vaccines require intricate manufacturing processes, which significantly extend production timelines. Furthermore, the potential presence of pre-existing antibodies against these microorganism vectors poses a substantial barrier to their implementation in personalized therapeutic strategies (49).

#### 2.1.3 Exosome-based vaccines

Exosome-based vaccines refer to tumor vaccines formulated with exosomes. Exosomes are tiny membrane vesicles secreted by various cell types, containing multiple bioactive molecules, and can transfer information between cells. Their immunomodulatory function depends on their cell origin (50–52). For example, exosomes derived from dendritic cells can enhance antigen presentation and T cell activation by carrying MHC I/II complexes, thereby strengthening the immune response. In contrast, tumor-derived exosomes have been implicated in immune suppression and tumor progression, reflecting their complex “double-edged sword” roles in cancer biology (53, 54). A Phase I clinical trial involving 40 patients with metastatic CRC evaluated the therapeutic potential of exosomes from tumor ascites (Aex) in combination with granulocyte-macrophage colony-stimulating factor (GM-CSF). The results demonstrated that the Aex combined with GM-CSF group elicited a stronger anti-tumor cytotoxic T-cell response, suggesting its promise as a safe and effective therapeutic vaccine (55). However, the therapeutic index of exosome-based vaccines is highly sensitive to manufacturing purity and standardization. One of the primary challenges is that vaccines derived from insufficiently purified tumor-associated exosomes may inadvertently carry immunosuppressive cargoes, thereby counteracting the intended anti-tumor response (56–58). Additionally, large-scale production and storage of exosomes in clinical applications continue to pose significant challenges (59). Although exosomes are generally regarded as biocompatible (60), their immunogenicity requires thorough evaluation, which substantially impacts their clinical utilization.

#### 2.1.4 Protein and peptide based vaccines

Peptide and protein vaccines are designed to trigger immune responses against specific tumor antigens. These vaccines mainly target immunogenic epitopes from tumor-associated antigens (TAAs) or TSA. Antigens are presented on the surface of APCs through MHC Class I or II molecules, activating T cells and inducing sustained antigen-specific immune memory (61). Generally, peptide cancer vaccines exhibit good tolerability among patients (62–65). A recent study demonstrated that PolyPEPI1018 combined with maintenance therapy is safe and well-tolerated in patients with microsatellite-stable (MSS) metastatic CRC (n=11). The vaccine induced CD8^+^ T-cell responses in 90% of patients, with 80% targeting at least three antigens, and achieved an objective response rate of 27.3%. Notably, two patients became eligible for curative surgery after vaccination, and those receiving multiple doses had significantly longer progression-free survival (12.5 vs. 4.6 months; P = 0.017), indicating promising clinical efficacy signals (63). In another investigation, the survivin-2B peptide vaccine was confirmed to be safe in HLA-A24-positive patients with advanced or recurrent colorectal cancer, with no severe adverse events reported. However, despite observed immunological responses—such as increased peptide-specific cytotoxic T lymphocytes (CTLs) frequency in one patient—the clinical benefits were modest: only transient tumor marker decreases in 6 out of 15 patients and a single minor response, while the majority showed disease progression (64). These findings underscore key limitations of peptide-based vaccines for personalized cancer immunotherapy. First, their strict HLA restriction limits applicability to specific patient subgroups (e.g., HLA-A24^+^ patients)—an inherent limitation of any epitope-based vaccine, but particularly pronounced in peptide vaccines due to their limited epitope capacity (typically 1–3 epitopes per peptide). Second, low intrinsic immunogenicity of short peptides often results in weak or transient T-cell activation, as reflected in the modest clinical responses observed. Unlike live or inactivated pathogens, peptides lack pathogen-associated molecular patterns (PAMPs) and fail to effectively stimulate innate immunity, necessitating the use of adjuvants, carrier proteins, or multimeric presentation systems (e.g., VLPs or nanoparticles) to enhance immunogenicity. Additionally, conformational limitations pose additional challenges for B-cell epitope design: linear peptides often fail to mimic native antigen structures, leading to antibodies that may not recognize the target protein in its natural conformation (66).

#### 2.1.5 DNA-based vaccines

DNA based vaccines utilize DNA as a template for encoding antigens, facilitating their transfection into cells. While DNA vaccines are generally less costly than RNA vaccines and exhibit greater stability, they have not yet been widely implemented in clinical practice (67). This is primarily due to the potential risk of genomic integration, which may lead to insertional mutagenesis, as well as the challenges posed by the prolonged expression of encoded antigens (68). Specifically, chronic exposure to the same antigen can induce immune tolerance or T-cell exhaustion, where the immune system ceases to recognize the vaccine-derived antigen as a foreign threat, thereby diminishing the therapeutic effect (69). Consequently, safety remains a significant concern regarding the application of DNA vaccines in CRC treatment.

In conclusion, while conventional vaccine approaches have demonstrated some progress in cancer therapy, they face common hurdles. These include limited immunogenicity, complex and time-consuming manufacturing processes for personalised products, and the constraints of immune compatibility (e.g., HLA restriction). Therefore, the development of a novel vaccine capable of addressing these interrelated challenges is critically imperative.

### 2.2 Structural and advantages of mRNA vaccine platforms

#### 2.2.1 Types and structural features of mRNA vaccines

**Figure 1:**
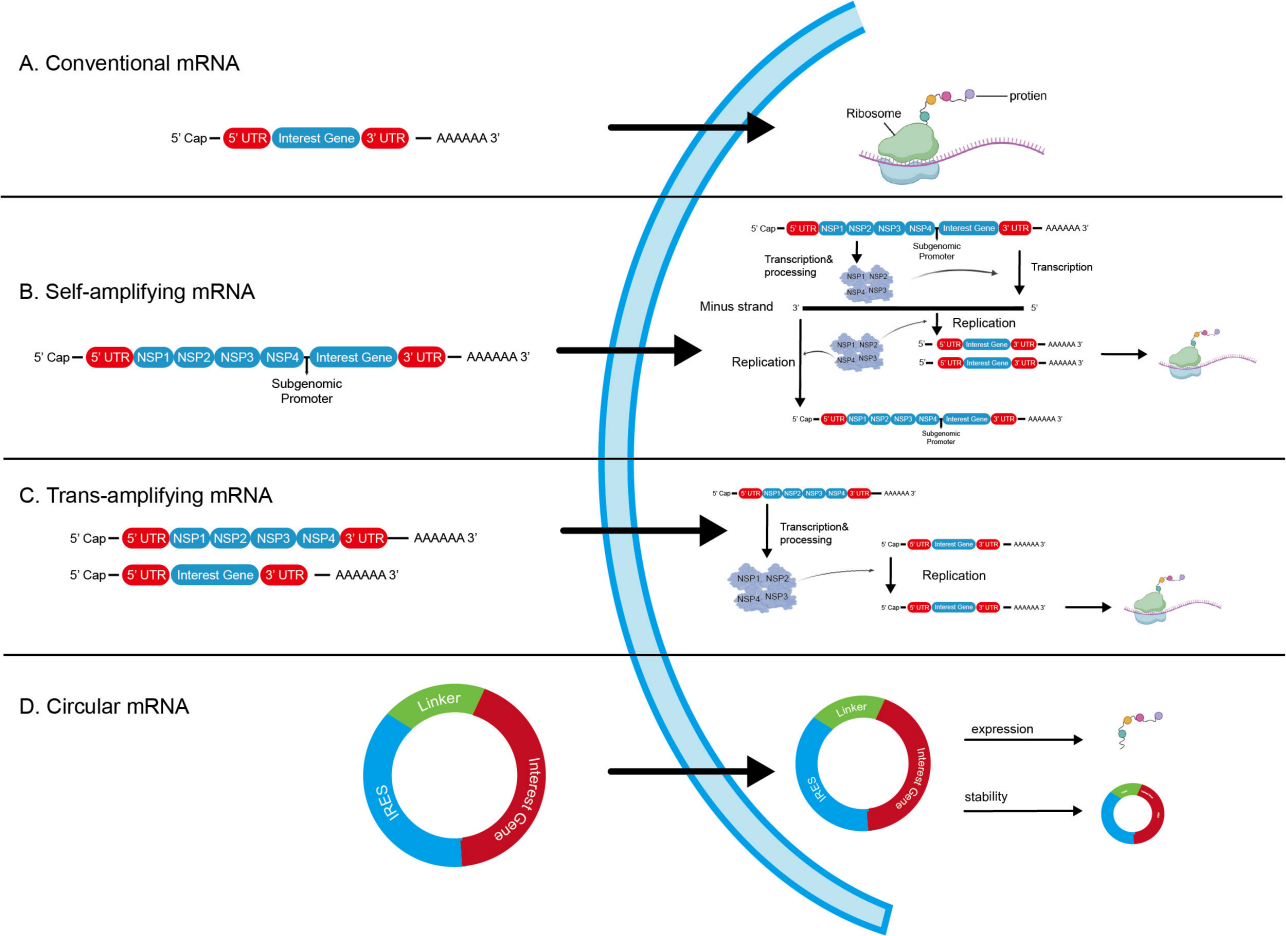
Schematic diagram of mRNA types. **(A)** conventional mRNA. **(B)**self-amplifying mRNA, which contains four genes encoding nonstructural proteins (NSP1–NSP4) that together constitute the replicase complex. Expression of the target gene is enabled by a regulatory subgenomic promoter. **(C)** trans-amplifying mRNA, consisting of two mRNA species: one retains the genes encoding the replicase, and the other expresses the gene of interest. **(D)** circRNA, composed of an IRES, interest gene, and a linking part—the linking part primarily refers to the circularization system/signal and other auxiliary elements (such as translation-enhancing elements). UTR, untranslated region; NSP, non-structural protein; IRES, internal ribosome entry site. Created with BioRender.com.

Both mRNA vaccines and traditional vaccines are designed to activate the immune system (70). Compared with traditional vaccine technologies, mRNA platforms offer distinct advantages in terms of production speed, mechanisms of action, safety profiles, and personalization capabilities (70, 71). Research has highlighted that these characteristics endow mRNA technology with significant therapeutic potential and strategic value in addressing the complexity and heterogeneity of cancer (71). Consequently, this technology has increasingly become a pivotal direction in the field of cancer vaccine research in recent years. Currently, there are three primary types of mRNA cancer vaccines: traditional non-replicating mRNA, self-amplifying RNA RNA (saRNA) and Circular RNAs (circRNAs) (**Figure 1**). The fundamental structure of traditional non-replicating mRNA consists of an open reading frame (ORF) region that encodes the target peptides sequence, flanked by five prime (5’) and three prime (3’) untranslated regions (UTR). This structure is further stabilized by a 7-methylguanylate (m7G) 5’ cap and a poly(A) tail at the 3’ end. The addition of the 5’ cap and 3’ poly(A) tail can occur during IVT or through enzymatic addition following initial IVT. In contrast, saRNA contains two ORFs; one encodes the targeted antigen sequence while the other encodes a viral replication mechanism that facilitates long-term RNA amplification within cells (72). Beissert et al. developed an enhanced version of saRNA termed trans-amplified RNA (taRNA) (73). This approach is based on a dual-vector system comprising two distinct templates, which separately generate antigen-encoding alphaviral RNA and replicase-encoding RNA separately. Compared to conventional saRNA, the binary design of taRNA allows for both simpler manufacturing and greater flexibility in functionalization. CircRNAs are widely expressed RNA transcripts found in different species, which enhances molecular stability by conferring resistance to exonuclease-mediated degradation (74–76).

CircRNAs can be genetically modified to facilitate protein synthesis by incorporating internal ribosome entry sites (IRES) or by including modified nucleotides, such as N^6^-methyladenosine, which can enhance translation efficiency (75). This structural resilience makes circRNAs particularly attractive for sustained antigen expression *in vivo*.

Upon cytosolic delivery, these mRNA constructs are translated by host ribosomes into antigenic proteins, which then undergo post-translational processing to yield functional, correctly folded immunogens. These structural characteristics enable mRNA vaccines to achieve efficient antigen expression and immune activation in CRC—an advantage of particular relevance in CRC, where inter- and intra-tumoral heterogeneity poses a major therapeutic challenge (11, 77).

#### 2.2.2 Advantages of mRNA vaccines

mRNA vaccines exhibit several significant advantages over conventional methods and may prove more effective against various cancers. First, the development of RNA-based vaccines is relatively rapid and cost-effective. IVT has transformed mRNA manufacturing through an advanced industrial system, significantly reducing production costs. The IVT process eliminates cellular and their associated regulatory obstacles, resulting in a more expedited production timeline compared to other vaccine modalities (78). Second, mRNA is non-infectious and non-integrative, which substantially reduces the risk of adverse immune responses while preventing the potential integration of foreign genes into the host genome (79). Furthermore, mRNA is transiently expressed as it is degraded by normal cellular mechanisms, enhancing its safety profile. Moreover, due to rapid advancements in various nucleotide modification techniques and improvements in *in vivo* delivery methods, contemporary mRNAs demonstrate enhanced stability and customizability while facilitating swift uptake and expression (80–82).

#### 2.2.3 Practical limitations and cross-platform comparison

Despite the transformative advantages of the mRNA platform, its practical limitations in the context of traditional vaccine strategies also warrant attention. These primarily include the following aspects:

(1) Delivery system: Precisely targeting intended cells (e.g., dendritic cells *in situ*) remains a primary challenge for ensuring vaccine efficacy (2). Response durability: While ensuring sustained protein expression, excessive reactogenicity must be tightly controlled to balance efficacy and safety (3). Manufacturing and logistics: Although production is rapid, the dependence on ultra-cold storage and process stability during scale-up remain bottlenecks for global deployment, especially in resource-limited regions.

Through the following table, a clear cross-platform comparison of key attributes of different vaccine platforms for CRC treatment can be provided. **Table 1** presents a summary of the advantages and disadvantages of different vaccine platforms for colorectal cancer.

**Table 1:** Comparison of colorectal cancer vaccines.

Vaccine platform	Subtype/personalization degree	Advantages	Disadvantages	References
Cell-Based Vaccines	tumour cell vaccines/Personalization	• Target a broad range of antigens • Induces both T- and B-cell responses	• Expensive, long production cycle, difficulty in tissue acquisition • Variable immunogenicity of antigens	(27, 29, 83)
	DC vaccines/Customizable	• Stimulates both CD4 ^+^ and CD8 ^+^ T cell responses • More durable immune responses	• Longer manufacturing time • Expensive and involves complicated cell culturing	(31, 37)
	allogeneic immune cell vaccine (AlloStim)/Non-personalized	• Off-the-shelf • Facilitates large-scale production and quality control	• Lack of specificity may prevent the induction of a strong adaptive immune response. • Risk of graft-versus-host disease or immune rejection response	(38, 39)
Exosome-based Vaccines	Personalization	• High safety, no cell vitality • Easy to store and transport	• Large-scale production and storage issues and standardization issues • Double-edged sword	(53, 54)
Protein and peptide based Vaccines	Customizable	• Straightforward GMP synthesis • Cheap, does not require cold-chain transportation	• Low immunogenicity; requires immune adjuvants to boost the immune response • The complexity of long peptide synthesis and purification leads to high HLA restriction.	(61, 63, 64)
Microorganism-based Vaccines	Bacterial vector vaccines/Customizable	• High immunogenicity and self-antigenicity • Can target tumor via intracellular infection	• Safety concerns due to live attenuated bacteria(such as systemic inflammation) • Complex production and manufacturing process	(47, 48)
	Viral vector vaccines/Customizable	• High immunogenicity and self-antigenicity • Can induce strong and durable immunity	• Pre-stored anti-vector antibodies may affect the therapeutic effect. • Safety issues (such as insertion mutations, excessive inflammatory responses)	(41, 42)
	Yeast vaccines/Customizable	• Stable and Cost-Effective • Can induce strong and durable immunity	• The production process and standardization still need to be improved. • Pre-stored antibodies may affect the therapeutic effect	(43–45)
DNA Vaccines	Customizable	• Lower production costs and degrade less readily vs mRNA neoantigen vaccines • Easy to encode multiple antigens in a single construct	• Low transfection efficiency, may require electroporation for efficient cellular uptake • Genomic integration risk	(69, 84)
mRNA Vaccines	Customizable	• Faster manufacturing time • Rapid induction of durable and functional CD8 ^+^ T cell responses • Easy to encode multiple antigens in a single construct	• Less stable, requires cold-chain logistics • Requires liposome or LNP for optimized delivery	(70, 85)

GMP, Good Manufacturing Practice; DC, Dendritic Cell; LNP, Lipid Nanoparticle; HLA, Human Leukocyte Antigen.

This comparison highlights that while mRNA technology offers unparalleled speed and flexibility for personalized therapy, challenges such as optimizing delivery systems for precise targeting(e.g., dendritic cells *in situ*) and ensuring durable protein expression without excessive reactogenicity remain critical hurdles for its widespread clinical application in CRC.

## 3 Mechanisms of mRNA vaccines in CRC

mRNA vaccines can treat tumors through at least two complementary mechanisms (1): remodeling the immunosuppressive tumor microenvironment (TME) to restore anti-tumor immunity; and (2) triggering specific immune responses to inhibit tumor growth (86, 87).

### 3.1 Tumor microenvironment of CRC

**Figure 2:**
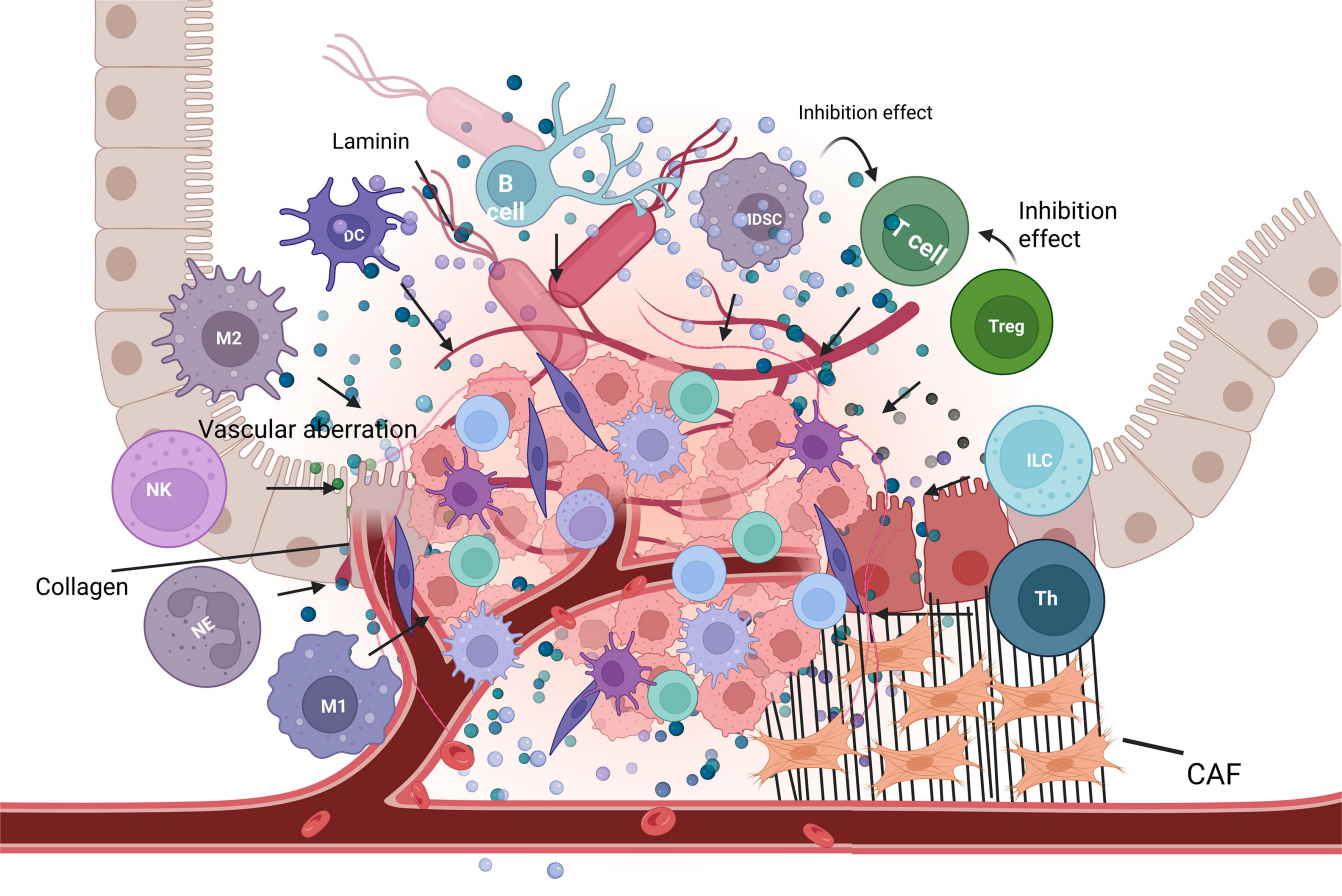
Schematic of the TME in CRC. The TME is composed of tumor cells, diverse immune cells (includingmacrophages, dendritic cells, lymphocytes, and neutrophils), cancer-associated fibroblasts (CAFs), the extracellular matrix (ECM), and aberrant tumor vasculature. Gut lumen-derived microbial components are localized near the tumor, implying their potential roles in tumor progression and immune modulation. The interactions among these cellular and non-cellular components constitute the complex TME. Created with BioRender.com.

The TME refers to the specific environment that tumor cells rely on for their survival (88). The living environment of tumor cells is illustrated in **Figure 2**. The TME comprises various components, including tumor cells, immune cells, Cancer-associated fibroblasts(CAFs), extracellular matrix, and cytokines. CRC cells facilitate tumor growth and promote immune evasion by regulating the cells and molecules that favor tumor development. For instance, TGF-β signaling is highly activated in the CMS4/MSS subtype, while a subset of CMS1/microsatellite instability-high (MSI-H) tumors also exhibits a TGF-β-dependent stromal signature (89–91). It is an important factor driving the functional heterogeneity of immune and stromal cells in CRC TME. TGF-β is one of the major stimuli that promoting to differentiation of Treg cells. These Treg cells predominantly inhibit CD8+ T cell activity in colorectal cancer (88, 92). In murine models of CMS4 CRC, tumor cells secrete TGF-β2 to activate tumor-associated neutrophils, which subsequently inhibit T cell activity (93). Furthermore, TGF-β induces a myofibroblast phenotype in CAFs, resulting in aberrant extracellular matrix (ECM) protein production that facilitates the exclusion of CD8+ T cells from the TME (94).

Hypoxia and metabolic alterations are two common phenomena that significantly impact the immune response in solid tumors, including CRC (95). As the tumor progresses, cells in the center region upregulate the expression of pro-angiogenic cytokines and hypoxia-inducible factors in response to hypoxic conditions, thereby accelerating tumor growth. This phenomenon directly impacts immune cell function within the TME; for instance, hypoxia promotes apoptosis of γδ T cells (96). This may be particularly relevant in the context of MSI-H CRCs with HLA class I defects, where γδ T cells have been demonstrated to serve as key effectors of immunotherapy by eliminating tumor cells that have lost MHC expression (97). Hypoxia also stimulates the secretion of VEGF and osteopontin by various cell types such as CAFs, contributing to intratumoral angiogenesis (98). Furthermore, structural and topological vascular abnormalities exacerbate intratumoral hypoxia. In both *in vitro* studies and mouse models of colorectal cancer, by-products of hypoxia, including lactate and metabolites derived from anaerobic glycolysis, exert broad tumor-promoting and immunosuppressive effects on CAFs, T cells, and bone marrow-derived cells (99–101). These factors impair tumor cell recognition by the immune system, thereby hindering the efficacy of tumor vaccines.

Given the pivotal role of the TME in CRC progression and immune evasion, targeting and reshaping this immunosuppressive milieu has become a critical therapeutic strategy. The inherent programmability of mRNA vaccines provides a versatile platform for this purpose, enabling precise TME modulation through multiple strategies—including direct immune modulation and the encoding of specific antigens, immunostimulatory factors. These approaches can collectively reverse immune suppression and activate potent anti-tumor immunity.

### 3.2 Activation of innate immunity by mRNA vaccines

mRNA vaccines have the potential to induce both innate and adaptive immunity, thereby exerting effective anti-tumor effects. They initiate innate immune responses by recognizing PAMPs via pattern recognition receptors (PRRs) (102). Upon entering the body, *in vitro*-synthesized mRNA and its delivery vectors are recognized as exogenous material by PRRs. This recognition activates the innate immune response. APCs produce pro-inflammatory cytokines and co-stimulatory molecules that attract and promote the infiltration of immune cells—such as T cells, natural killer (NK) cells, basophils, and macrophages—into the tumor microenvironment (103). By recruiting key immune cells, this process drives a sustained antitumor immune response and the generation of adaptive B and T cell immunity.

Furthermore, the immunogenicity of mRNA is primarily mediated by Toll-like receptor 7 (TLR7) and TLR8 (11). TLR7 is expressed on B cells, macrophages, and DCs, where it functions to detect single-stranded RNA (ssRNA). TLR7 signaling enhances the production of pro-inflammatory cytokines and antigen presentation, while also improving the survival of memory B cells (104). Additionally, the myeloid differentiation factor 88 (MYD88)/TLR7 pathway drives the type I interferon (IFN-I) response and promotes a pro-inflammatory state via cytokine secretion (105). This pathway’s stimulatory effects upregulate the adaptive immune response induced by mRNA vaccines, and it also mediates B cell activation (106, 107). Vaccines encoding the B cell epitope trigger a specific antimetastatic effect (18). In contrast, TLR8 is mainly highly expressed in myeloid immune cells, including monocytes, myeloid dendritic cells and neutrophils (108). Similar to TLR7, TLR8 can also recognize ssRNA and its degradation products (such as fragments rich in GU sequences), thereby sensing the entry of exogenous mRNA vaccines. After activation, TLR8 also relies on MYD88 for signal transduction, initiating downstream NF-κB and MAPK pathways, and inducing pro-inflammatory cytokines (such as TNF-α, IL-12) (109, 110). It is noteworthy that the activation of TLR8 not only directly promotes the maturation of antigen-presenting cells but also, through synergy with TLR3/4, shapes an immune microenvironment biased towards Th1 type (111, 112), thereby enhancing the response of cytotoxic T cells. Additionally, the TLR8 signal may also play a certain role in regulating the inhibitory function of regulatory T cells (Treg cells), thereby indirectly influencing the intensity and persistence of the immune response induced by the vaccine (112).

In summary, these mechanisms demonstrate that mRNA vaccines can modulate acquired immunity and the tumor microenvironment through the regulation of innate immune pathways.

### 3.3 mRNA vaccines encoding tumor-associated antigens

TAAs are antigenic molecules found on both tumor cells and normal cells, including embryonic proteins, glycoprotein antigens, squamous cell antigens, among others (113). TAAs are commonly utilized in the clinical diagnosis of tumors; however, they are not exclusively characteristic of tumor cells. Normal cells can also synthesize these antigens in trace amounts, and their expression levels significantly increase during the proliferation of tumor cells. Most mammals demonstrate a significant degree of immune tolerance to TAAs, presenting a major challenge for the development of cancer vaccines that leverage these antigens due to central immune tolerance mechanisms (114). The trend in developing clinical targeted mRNA cancer vaccines has shifted towards employing multiple combinations of shared TAAs (115). When the mRNA encoding neoantigen enters the body, it will produce antigen proteins in the target cells. These proteins are processed into peptide epitopes that bind to MHC-I molecules. The resulting peptide–MHC-I complexes are presented to CD8^+^ T cells, activating them to differentiate into CTLs that directly kill antigen-expressing tumor cells (116). Additionally, secreted or released antigens can be taken up and presented by antigen-presenting cells via the MHC class II pathway. CD4^+^ T cells recognize these peptide–MHC-II complexes and differentiate into helper T cells, which support and modulate the ongoing immune response (117).

CEA is the most common TAA, expressed in nearly all colorectal cancers. Therefore, it represents a highly attractive option in clinical immunotherapy protocols. Multiple clinical studies have demonstrated that DCs loaded with CEA peptides can induce antigen-specific T cell responses in patients with colorectal cancer (23, 118, 119). Consequently, the use of CEA mRNA vaccines may theoretically elicit a broader and more robust T cell repertoire due to the expression of multiple epitopes. In other cancers, CEA has already shown clinical efficacy. For instance, several clinical trials have demonstrated that mRNA vaccines encoding TAAs can induce T cell responses in tumors such as melanoma (NCT04526899, NCT01278940, NCT01995708).

However, the results of TAA-based mRNA vaccines in CRC have been mixed. In a Phase I/II clinical trial (NCT00228189) involving CRC (35), peripheral blood mononuclear cells subjected to mRNA electroporation were used as stimulator cells and co-cultured with T cells. In the DTH skin test, CEA peptide-specific T cell reactivity was observed in 8 patients; conversely, no CEA peptide specificity was detected in 5 patients who received mRNA inoculation. This discrepancy may be attributed to a combination of factors including the patient’s baseline immune status, antigenic characteristics, vaccine delivery efficiency, and the sensitivity of the detection method employed. Further studies are warranted to assess the application of mRNA encoding TAAs, such as CEA mRNA, in CRC.

### 3.4 mRNA vaccines encoding tumor-specific antigens

TSAs are uniquely expressed on tumor cells and absent from normal tissues. Targeting TSAs represents a pivotal strategy in cancer immunotherapy, where the selection of appropriate antigens is critical for vaccine efficacy. Unlike TAAs, which are susceptible to central and peripheral immune tolerance, TSAs—particularly neoantigens arising from somatic mutations—exhibit high immunogenicity and can effectively overcome these tolerance barriers (120). Its mechanism of action within the body is the same as that described in Section 3.3, whereby antigen presentation leads to the activation of both CD8^+^ and CD4^+^ T cells, eliciting anti-tumor responses. Through these mechanisms, antigens encoded by mRNA vaccines targeting multiple TSAs can be presented through both the MHC I and MHC II pathways, thereby inducing a broad polyclonal immune response and effectively reducing immune escape caused by antigen loss (although it is important to acknowledge that these fundamental biological limitations cannot be completely eliminated) (121, 122). Preclinical evidence supports this approach. For instance, Zhang et al. developed a neoantigen−mRNA/DC vaccine using candidate neoantigens from mouse colon cancer (MC38) and evaluated its immune and antitumor effects (20). The results demonstrated that the neoantigen−mRNA/DC vaccine induced strong T cell immune responses and exhibited significant antitumor effects, effectively preventing tumor growth. A Phase I trial reported that three out of four patients treated with the neoantigen-targeting mRNA-4650 vaccine developed vaccine-induced CD8^+^ and CD4^+^ T-cell responses specific to the encoded neoantigens (5). Despite the limitations of a small sample size and tumor heterogeneity, the high response rate observed in three out of four cases preliminarily validates the technical feasibility of the TSA mRNA vaccine platform. These findings also underscore the necessity of utilizing biomarkers to identify patient subgroups in larger subsequent trials, thereby advancing the development of precision immunotherapy.

### 3.5 mRNA vaccines encoding immunostimulatory factors

Beyond delivering antigens, mRNA technology offers the flexibility to encode immunostimulatory factors that potentiate vaccine-induced antitumor immunity. While mRNA-encoded immunostimulatory factors are also being explored as standalone immunotherapies for direct intratumoral injection, this section focuses on their role in mRNA vaccine formulations, where they are co-delivered with TAAs or TSAs to enhance and sustain immune responses. By encoding immunomodulatory proteins (e.g., cytokines, co-stimulatory ligands, receptors, or enzymes), these vaccine components directly activate immune function and strengthen the body’s defense against tumors (115). A key advantage of mRNA technology in vaccine design is the ability to co-deliver multiple immunostimulatory sequences alongside antigen-encoding sequences, thereby activating synergistic pathways that amplify antigen-specific immunity. The most commonly utilized cytokines include Interleukin-2 (IL-2), IL-12, and OX40 Ligand (123). Although initially developed as intratumoral immunotherapies, studies of IL-12 mRNA have provided mechanistic insights relevant to vaccine development. For instance, in a Phase I trial of an IL-12 mRNA (NCT05392699), dose-dependent increases in C-X-C Motif Chemokine Ligand 9 (CXCL9), CXCL10, and CXCL11 levels were observed in peripheral blood samples, indicating activation of the IFN-γ downstream signaling pathway. By day 7 post-treatment, increased CD8^+^ T cell infiltration and elevated Programmed Death-Ligand 1 (PD-L1) expression were observed within the patients’ TME (124). These findings suggest that incorporating immunostimulatory factors such as IL-12 into mRNA vaccines could help reverse local immunosuppression and enhance the presentation of vaccine-encoded antigens.

Notably, mRNA-encoded immunostimulatory factors can also reprogram the TME by polarizing tumor-associated macrophages (TAMs) from a pro-tumorigenic M2 state toward an anti-tumor M1 phenotype (125). M1-like TAMs promote anti-tumor immunity by activating T cells, producing inflammatory cytokines, and enhancing phagocytosis (126). Within vaccine formulations, these immune-stimulating factors function as potent molecular adjuvants, augmenting both the innate immune response and the antigen-specific adaptive response triggered by TAAs or TSAs. The presence of these immune-stimulating factors can further amplify the immune response triggered by TAAs/TSAs, facilitating more effective recognition and targeting of tumor cells by the immune system. For example, in a preclinical study, combining tumor lysate with an IL-23A mRNA vaccine significantly promoted systemic immune activation and demonstrated promising efficacy in murine models of CT26 colon carcinoma abdominal and lung metastasis (127).

## 4 Current clinical translational status and combination therapy strategies

Clinical translational research on mRNA vaccine therapies for solid tumors is advancing expeditiously, with explorations in the field of CRC also exhibiting a trend toward diversification.mRNA-based therapeutic approaches are increasingly emerging as a promising strategy to combat tumor immune evasion. However, the scientific rigor of clinical trial design and safety management, alongside tumor heterogeneity and immunosuppressive TME in CRC, remain significant current challenges. mRNA-based therapeutic approaches are increasingly emerging as a promising strategy to combat tumor immune evasion. However, the scientific rigor of clinical trial design and safety management, alongside tumor heterogeneity and immunosuppressive TME in CRC, remain significant current challenges (77, 128, 129). The diversity of ongoing clinical trials is summarized in the table below **Table 2**.

**Table 2:** The ongoing clinical trial involving mRNA vaccines for colorectal cancer.

NCT number	Platform	Antigen type	Combinatorial agent(s)	Phase	Enrollment (Estimated)	Primary endpoint	Status
NCT06497010	Traditional mRNA	Personalized neoantigen	PD-1 inhibitors	Early Phase 1	40	RP2D	Recruiting
NCT06577532	Traditional mRNA	Mutant KRAS neoantigens	Toripalimab	Early Phase 1	56	DLT,safety,ORR	Recruiting
NCT07182435	Traditional mRNA	Personalized neoantigen	/	Early Phase 1	36	DLT, safety, Immunogenicity of a personalized cancer vaccine	Not yet recruitingecruiting
NCT05359354	Traditional mRNA	Personalized neoantigen	PD-1 inhibitors	Not Applicable	36	MTD, DLT, Safety	Unknown status
NCT05940181	Traditional mRNA	Personalized neoantigen	Sintilimab	Not Applicable	9	MTD, DLT, Safety	Unknown status
NCT05949775	Traditional mRNA	Personalized neoantigen	Stintilimab	Not Applicable	20	PFS	Not yet recruiting
NCT06195384	Traditional mRNA	Personalized neoantigen	/	Phase 1	30	DLT	Recruiting
NCT05942378	Traditional mRNA	Personalized neoantigen	Adebrelimab	Phase 1	30	RP2D, Reaction of antigen-specific T cells	Unknown status
NCT07067385	Traditional mRNA	Personalized neoantigen	Stinlimab	Phase 1	40	Safety, Immunogenicity of a personalized cancer vaccine	Recruiting
NCT07245901	circmRNA	FAM53B-219aa	Toripalimab	Phase 1,2	60	Safety, RP2D, MTD	Not yet recruiting
NCT05141721	self-amplifying mRNA	Personalized neoantigen	heterologous chimpanzee adenovirus vaccine, Atezolizumab, Ipilimumab, Fluoropyrimidine plus leucovorin, Bevacizumab	Phase 2,3	700	ctDNA, PFS	Active, not recruiting

RP2D, recommended phase 2 dose; DLT, dose-limiting toxicity; ORR, objective response rate; MTD, maximum tolerated dose; ctDNA, circulating tumor DNA; PFS, progression-free survival.

### 4.1 Patient stratification: biomarkers for predicting CRC vaccine responsiveness

Personalized vaccines targeting tumor neoantigens are one of the core directions (such as NCT06195384, NCT05949775, etc.). This trend reflects the concept of precision medicine, but it also faces technical challenges such as standardization of neoantigen screening, HLA restriction analysis, and clonal assessment (130). In terms of clinical trial design, such studies usually require the use of composite endpoints: in the early stage of exploration, the main focus is on safety-related indicators (such as maximum tolerated dose, dose-limiting toxicity) (131). Furthermore, traditional response criteria—morpho- logical (such as RECIST), often fail to accurately and timely capture the therapeutic efficacy of immunotherapy, including personalized vaccines (132). Therefore, the application of alternative endpoints such as circulating tumor DNA (ctDNA) and immunogenicity of a personalized cancer vaccine (through ELISpot or TCR sequencing) as composite endpoints to precisely evaluate effectiveness has also become inevitable.

Secondly, combination therapy has been widely adopted, with its theoretical basis lying in the simultaneous activation of the immune system and the removal of its inhibitory mechanisms. Preliminary clinical studies have shown its potential (133, 134). In a study involving patients with advanced metastatic solid tumors (NCT03639714), a personalized vaccine regimen combining a chimpanzee adenovirus and a saRNA neoantigen vaccine was found to be safe and well-tolerated. Furthermore, improved overall survival (OS) was observed in several patients with MSS CRC (133). mRNA-5671/V941, administered either as a monotherapy or in combination with pembrolizumab, exhibited no dose-limiting toxicities (DLTs) in patients with colorectal cancer (NCT03948763), with signs of immune activation observed in a subset of participants. However, the majority of patients experienced varying degrees of adverse events, highlighting the complexity of its safety profile^4^. Consequently, how to distinguish the contributions of mRNA and other drugs such as immune checkpoint inhibitors (ICI) and how to avoid the superimposition of toxicity remain core issues (135, 136). To clarify the source of efficacy, a three-arm trial design (ICI monotherapy, vaccine monotherapy, and combination therapy) may be needed, or by tracking the clonal expansion of vaccine-specific T cells, to attribute the efficacy signal (137). The safety management of combination therapy is of vital importance. Based on the current clinical trial results, the background risks of mRNA vaccines themselves (such as fever and injection site reactions) are usually controllable (138). However, when combined with ICI, the theoretical risk of immune-related adverse events (irAEs) increases (135). Therefore, clear management strategies need to be formulated: closely monitor high-risk patients, establish irAE intervention plans, and explore preventive measures. Optimizing the timing of vaccination is key to enhancing therapeutic efficacy. For patients with postoperative minimal residual disease, the immune system has not yet been suppressed by advanced tumor burden, making it an ideal window for vaccination (139). Additionally, Grippin and colleagues demonstrated that SARS-CoV-2 mRNA vaccines can temporarily reset the tumor-immune interface, transforming “cold” tumors into those responsive to Programmed Cell Death Protein 1 (PD-1)/PD-L1 blockade (140). This could be a crucial step towards precision oncology, with treatment designs based on “opportunity windows” centered around the combined use of mRNA vaccines and immune checkpoint inhibitors (19).

Third, novel platforms continue to emerge, with circular RNA-based vaccines (e.g., circFAM53B, NCT07245901) representing an innovative approach to enhance antigen expression stability and durability. This technological breakthrough is expected to achieve more sustained antigen expression and stronger immune memory (141–143). However, the potential for conditional immune activation raises concerns about their safety and controllability (144). Early trials need to closely monitor excessive inflammatory responses such as cytokine storms and assess long-term risks.

In summary, the current trend in mRNA vaccine clinical trials is not only reflected in technological iterations but also in the in-depth consideration of design scientificity and safety. Future research should integrate biomarker stratification, precise endpoint selection, reasonable timing arrangements, and proactive toxicity management to promote the clinical translation of this therapy in colorectal cancer (145).

### 4.2 Biomarkers of vaccine reactivity in colorectal cancer and patient stratification

The clinical efficacy of mRNA vaccines in triggering anti-tumor immunity varies among different types of colorectal cancer patients. This heterogeneity highlights the importance of predictive biomarkers in patient selection and in guiding the development of rational combined treatment regimens (88).

#### 4.2.1 MSI-H/dMMR versus MSS/pMMR

MSI-H/mismatch repair-deficient (dMMR) and MSS/proficient Mismatch Repair (pMMR) are the most fundamental molecular subtypes in colorectal cancer, which have a profound impact on clinical immunotherapy (146).

MSI-H/dMMR CRC, encompassing approximately 15% of all cases (with a higher prevalence in early-stage and right-sided tumors), is characterized by a defective DNA mismatch repair system (146). MSI-H/dMMR type colorectal cancer, due to its high antigen load and immune infiltration characteristics, is considered a promising candidate population for mRNA vaccine strategies (19).

In addition, the TME of MSS CRC typically exhibits immunosuppressive characteristics. Nevertheless, these hurdles do not preclude the therapeutic potential of vaccine-based strategies. Emerging clinical data suggest that mRNA-based platforms, particularly when targeting individualized neoantigens, hold promise for this patient population (133). By remodeling the TME, such integrated strategies can synergize with vaccines to elicit durable and robust immune responses.

Therefore, MSI status plays a crucial screening role in patient stratification. However, it must be acknowledged that there is heterogeneity within the MSI groups. For instance, MSH2/MSH6-deficient tumors typically exhibit a higher average Tumor mutational burden (TMB) than MLH1/PMS2-deficient tumors; MSS tumors with POLE/POLD1 mutations have an ultra-high mutation phenotype (usually over 100 mutations per megabase) (147, 148). This heterogeneity within the MSI groups further highlights the importance of other biomarkers, such as absolute TMB values, clonality of neoantigens, or specific genetic drivers of MMR deficiency, in optimizing patient selection and the efficacy of personalized vaccine approaches.

#### 4.2.2 Tumor mutation burden, neoantigen quality, and HLA constraints

TMB provides a continuous and quantitative measure of genomic instability, which can further refine the aforementioned stratification. TMB, typically defined as the total number of somatic nonsynonymous mutations per megabase, serves as a proxy for the potential neoantigen pool (149). However, the relationship between TMB and vaccine responsiveness is not linear; a high mutational burden does not guarantee the presence of immunogenic epitopes, nor does it cover the complex interactions between antigen presentation and immune evasion. Therefore, the entire process from antigen generation to presentation needs to be considered. Evidence suggests that a high mutational burden does not consistently predict the presence of immunogenic epitopes, nor does it account for the complex interplay between antigen presentation and immune evasion mechanisms (150). Therefore, a multidimensional assessment of the entire process—spanning from antigen generation to presentation—is essential to better understand and predict vaccine efficacy.

The clonality and immunogenicity of neoantigens are also crucial for effectively activating immune responses (151). CRC exhibits extensive branched evolutionary features, with scarce clonal neoantigens and mainly subclonal neoantigens (152). Clonal neoantigens can drive complete tumor clearance; while subclonal neoantigens are prone to causing immune escape (153). In addition, the prediction of the immunogenicity of neoantigens and the actual expression of the mutated genes is also crucial for ensuring that neoantigens can be truly presented. The neoantigens expressed by mRNA vaccines are presented by HLA. However, approximately 21% to 28% of colorectal cancer patients have HLA-Loss of heterozygosity (HLA-LOH), which leads to the interruption of the antigen presentation pathway (153). This is also associated with a large number of subclonal neoantigens (154). Therefore, it is necessary to verify the quality of MHC expression, and ideally, new epitopes should be selected based on the corresponding MHC expression.

#### 4.2.3 Immunosuppressive features of the tumor microenvironment

CRC TME usually has immunosuppressive effects, and its complex composition determines the efficacy of vaccine-induced T cell responses.From the perspective of the transcriptome, CMS classification further refines the immune phenotype of the TME. For instance, in CMS1 tumors, there is a dense infiltration of T cells, but the high expression of checkpoints such as PD-1 and TIM-3 reflects the exhausted state of T cells (155), suggesting the necessity of combining ICI when applying mRNA vaccines. In the CMS4 subtype, activated CAFs deposit a large amount of extracellular matrix, and patients with CAF enrichment may benefit from TGF-β inhibitors or matrix remodeling drugs (156). Mechanistically, this can collaborate with mRNA vaccines. In addition, CMS2/3 tumors are typically classified as immune desert subtypes with minimal T cell infiltration. For such “cold” tumors, inducing immunogenic cell death via chemotherapy could be a crucial step to transform them into “hot” tumors, thereby enhancing the efficacy of subsequent vaccination (157). It is also important to note that most CRC tumors exhibit intratumoral heterogeneity, which highlights the significance of multi-point dynamic detection and more precise TME biomarkers (such as T-cell exhaustion scores and CAF enrichment levels) in the clinical application of mRNA vaccines and combination therapy (158, 159).

## 5 Future directions

mRNA vaccines have great potential in the field of immunotherapy for colorectal cancer. However, further research and clinical trials are essential to optimize their efficacy, safety, and long-term benefits. Potential future directions and advancements include:

### 5.1 Optimizing mRNA vaccine design

mRNA can induce cytokine production by activating innate immune responses. However, excessive or sustained cytokine release may lead to severe side effects, including autoimmune reactions, and potentially interfere with specific immune responses against vaccine antigens (160). Excessive innate sensing may impair translation and shorten expression duration, whereas excessive suppression of intrinsic immunostimulatory signals may weaken dendritic cell activation and T-cell priming (161). Therefore, next-generation mRNA design should focus on fine-tuning, rather than eliminating, innate immune stimulation. Optimization at the RNA level should extend beyond nucleoside substitution to include coordinated engineering of the 5′ cap, untranslated regions, poly(A) tail, codon usage, and RNA secondary structure, with the goal of controlling not only expression intensity but also expression kinetics and immunological outcome. Additionally, the IVT preparation process often generates double-stranded RNA (dsRNA) by-products (162). These dsRNA impurities can activate intracellular immune sensing pathways, such as upregulating protein kinase R and oligoadenylate synthase, thereby triggering IFN-I-mediated immune responses that lead to rapid degradation of mRNA and compromise vaccine efficacy (163). While the removal of dsRNA via High-Performance Liquid Chromatography (HPLC) or RNase III enzymatic digestion is technically feasible in industrial settings, these downstream purification methods face significant challenges in terms of scalability, high operational costs (163). Therefore, future research should prioritize upstream process optimization—such as refining IVT parameters and engineering more precise RNA polymerases—to mitigate dsRNA formation at the source, thereby enhancing both the purity and potency of mRNA vaccines. Finally, delivery systems should be optimized not only to protect mRNA from degradation, but also to improve tissue distribution, antigen-presenting cell targeting, repeated-dose tolerability, and safety (164). Thus, future advances will likely depend on integrated strategies that combine RNA engineering, immune modulation, and precision delivery to generate more potent and clinically translatable mRNA cancer vaccines.

### 5.2 Innovations in mRNA vaccine storage methods

The development of new technologies is crucial for stabilizing vaccines while addressing some limitations associated with traditional freeze-dried storage (78). Spray drying and freeze-drying technologies provide a practical technical path for the room-temperature stable storage of mRNA preparations (165). For example, mRNA-1273 can be stored in a -20 °C freezer for 6 months, and can be kept in a refrigerated environment at 2 °C to 8 °C for one month (166). The focus of future research will be on optimizing the formulation to further extend the preservation of mRNA. In particular, the development of non-freezing liquid formulations will have a profound impact on the widespread availability of mRNA vaccine preparations in resource-poor regions. In addition, the establishment of a long-term stability evaluation system in the dried state, as well as the development of universal freeze-drying processes for different mRNA sequences (such as self-amplifying mRNA), will also be important directions for subsequent research.

### 5.3 The choice of injection route

The injection route is crucial for the translation efficiency of target proteins and the distribution of mRNA-based cancer vaccine. Intravenous injection remains the most common route for mRNA-based cancer vaccines in current clinical trials and effectively targets multiple lymphoid organs. However, this method may also lead to off-target effects such as systemic inflammatory responses (167). Meanwhile, subcutaneous administration is widely used in early-stage cancer RNA vaccines, such as CV9103. This approach utilizes the high density of dendritic cells and abundant lymphatic vessels in the skin to induce an effective immune response, but it may induce local side effects (168). Preclinical and clinical studies have explored various injection routes, including intravenous, subcutaneous, intradermal, intratumoral, and intranodal injection. Nevertheless, comprehensive experiments are still needed to fully exploit the great potential of mRNA-based cancer vaccines.

### 5.4 Combination therapy

The combined design of mRNA vaccines and immunotherapy has shown initial success. Through the combination of molecular pathways and immunodynamics, synergy is achieved, such as mRNA vaccines inducing the “interferon window” through type I interferons to synergize with the combination of immune checkpoint inhibitors, achieving the maximum efficacy (140). Future research should focus on clinical validation and combined administration under multimodal temporal schemes.

### 5.5 Biomarker development

As the clinical research on CRC mRNA vaccines progresses, future studies should focus on the dynamic biomarkers of the immune response driven by the vaccine. Since this therapy exerts its anti-tumor effect by activating the immune system, its clinical efficacy may not be reflected by conventional imaging evaluations (such as possible pseudo-progression (where the tumor temporarily enlarges) or good therapeutic effects observed in cases with a relatively mild disease burden but without obvious imaging remission) (169). For instance, ctDNA is a non-invasive and sensitive dynamic monitoring marker; a decline in its levels during treatment has shown potential correlation with prolonged overall survival, especially helpful for precisely stratifying molecularly remitted patients with stable disease as shown by traditional imaging (133). Biomarkers facilitate patient stratification, treatment monitoring, and assessment of treatment response, thereby enabling personalized treatment strategies.

## 6 Conclusions

With the implementation of numerous clinical trials, mRNA vaccines have shown promising early-stage results, demonstrating their safety, feasibility, and potential efficacy in the treatment of CRC. As a modular and customizable platform, these vaccines can elicit tumor-specific responses through various pathways while also modulating the tumor microenvironment. This positions them as a highly promising therapeutic approach. In conclusion, continuous research and progress in this field are expected to transform CRC immunotherapy, providing patients with more effective and personalized treatment options.

## Footnotes

1 Clinical trial registration: US Clinical Trial Database (ClinicalTrials.gov), registration number: NCT01505166. https://clinicaltrials.gov/study/NCT01505166

2 Clinical trial registration: US Clinical Trial Database (ClinicalTrials.gov), registration number: NCT02380443, NCT01065441, NCT00861107. https://clinicaltrials.gov/study/NCT02380443; https://clinicaltrials.gov/study/NCT01065441; https://clinicaltrials.gov/study/NCT00861107;

3 Clinical trial registration: US Clinical Trial Database (ClinicalTrials.gov), registration number: NCT02380443. https://clinicaltrials.gov/study/NCT02380443

4 Clinical trial registration: US Clinical Trial Database (ClinicalTrials.gov), registration number: NCT03948763. https://clinicaltrials.gov/study/NCT03948763

## Glossary

CRC: Colorectal cancer
mRNA: messenger RNA
IVT: in vitro transcription
APCs: antigen-presenting cells
HLA: human leukocyte antigen
DC: dendritic cell
DTH: delayed-type hypersensitivity
CEA: carcinoembryonic antigen
IFN-γ: interferon-gamma
Aex: exosomes from tumor ascites
GM-CSF: granulocyte-macrophage colony-stimulating factor
MHC: major histocompatibility complex
TME: tumor microenvironment
VEGF: vascular endothelial growth factor
CAFs: tumor-associated fibroblasts
TGF: transforming growth factor
NK cell: natural killer cell
TAAs: tumor-associated antigens
TSAs: tumor-specific antigens
TAMs: tumor-associated macrophages
CTLs: cytotoxic T lymphocytes
saRNA: Self-amplifying RNA
circRNA: Circular RNA
MSS: microsatellite-stable
taRNA: trans-amplified RNA
MSI-H: Microsatellite Instability-High
PAMP: pathogen-associated molecular pattern
PRR: pattern recognition receptor
TLR7: Toll-like receptor 7
ssRNA: single-stranded RNA
MYD88: myeloid differentiation factor 88
Treg: regulatory T cells
ICI: immune checkpoint inhibitors
irAE: immune-related adverse events
dMMR: mismatch repair-deficient
pMMR: proficient Mismatch Repair
TMB: Tumor mutational burden
PD-1: Programmed Cell Death Protein 1
PD-L1: Programmed Death-Ligand 1
CXCL9: C-X-C Motif Chemokine Ligand 9
IL-2: Interleukin-2
LNP: lipid nanoparticles
ctDNA: circulating tumor DNA

## References

[1] Bray F Laversanne M Sung H Ferlay J Siegel RL Soerjomataram I . Global cancer statistics 2022: GLOBOCAN estimates of incidence and mortality worldwide for 36 cancers in 185 countries. CA Cancer J Clin. (2024) 74:229–63. doi:10.3322/caac.21834. PMID: 38572751

[2] Keum N Giovannucci E . Global burden of colorectal cancer: emerging trends, risk factors and prevention strategies. Nat Rev Gastroenterol Hepatol. (2019) 16:713–32. doi:10.1038/s41575-019-0189-8. PMID: 31455888

[3] Song M Huang S Wu X Zhao Z Liu X Wu C . UBR5 mediates colorectal cancer chemoresistance by attenuating ferroptosis via Lys 11 ubiquitin-dependent stabilization of Smad3-SLC7A11 signaling. Redox Biol. (2024) 76:103349. doi:10.1016/j.redox.2024.103349. PMID: 39260061 PMC11415886

[4] Antoniotti C Rossini D Pietrantonio F Catteau A Salvatore L Lonardi S . Upfront FOLFOXIRI plus bevacizumab with or without atezolizumab in the treatment of patients with metastatic colorectal cancer (AtezoTRIBE): a multicentre, open-label, randomised, controlled, phase 2 trial. Lancet Oncol. (2022) 23:876–87. doi:10.1016/S1470-2045(22)00274-1. PMID: 35636444

[5] Cafri G Gartner JJ Zaks T Hopson K Levin N Paria BC . mRNA vaccine–induced neoantigen-specific T cell immunity in patients with gastrointestinal cancer. J Clin Invest. (2020) 130:5976. doi:10.1172/JCI134915. PMID: 33016924 PMC7598064

[6] Van Hoecke L Van Lint S Roose K Van Parys A Vandenabeele P Grooten J . Treatment with mRNA coding for the necroptosis mediator MLKL induces antitumor immunity directed against neo-epitopes. Nat Commun. (2018) 9:3417. doi:10.1038/s41467-018-05979-8. PMID: 30143632 PMC6109072

[7] Moore ZS Seward JF Lane JM . Smallpox. Lancet. (2006) 367:425–35. doi:10.1016/S0140-6736(06)68143-9. PMID: 16458769

[8] Kantoff PW Higano CS Shore ND Berger ER Small EJ Penson DF . Sipuleucel-T immunotherapy for castration-resistant prostate cancer. N Engl J Med. (2010) 363:411–22. doi:10.1056/NEJMoa1001294. PMID: 20818862

[9] Zaidi N Jaffee EM Yarchoan M . Recent advances in therapeutic cancer vaccines. Nat Rev Cancer. (2025) 25:517–33. doi:10.1038/s41568-025-00820-z. PMID: 40379970

[10] Sharpless NE . COVID-19 and cancer. Science. (2020) 368:1290. doi:10.1126/science.abd3377. PMID: 32554570

[11] Deng Z Tian Y Song J An G Yang P . mRNA vaccines: the dawn of a new era of cancer immunotherapy. Front Immunol. (2022) 13:887125. doi:10.3389/fimmu.2022.887125. PMID: 35720301 PMC9201022

[12] Wolff JA Malone RW Williams P Chong W Acsadi G Jani A . Direct gene transfer into mouse muscle *in vivo*. Science. (1990) 247:1465–8. doi:10.1126/science.1690918. PMID: 1690918

[13] Gote V Bolla PK Kommineni N Butreddy A Nukala PK Palakurthi SS . A comprehensive review of mRNA vaccines. Int J Mol Sci. (2023) 24:2700. doi:10.3390/ijms24032700. PMID: 36769023 PMC9917162

[14] Karikó K Muramatsu H Welsh FA Ludwig J Kato H Akira S . Incorporation of pseudouridine into mRNA yields superior nonimmunogenic vector with increased translational capacity and biological stability. Mol Ther. (2008) 16:1833–40. doi:10.1038/mt.2008.200. PMID: 18797453 PMC2775451

[15] Sahin U Derhovanessian E Miller M Kloke B-P Simon P Löwer M . Personalized RNA mutanome vaccines mobilize poly-specific therapeutic immunity against cancer. Nature. (2017) 547:222–6. doi:10.1038/nature23003. PMID: 28678784

[16] Sette A Vitiello A Reherman B Fowler P Nayersina R Kast WM . The relationship between class I binding affinity and immunogenicity of potential cytotoxic T cell epitopes. J Immunol. (1994) 153:5586–92. doi:10.4049/jimmunol.153.12.5586 7527444

[17] Gurung HR Heidersbach AJ Darwish M Chan PPF Li J Beresini M . Systematic discovery of neoepitope–HLA pairs for neoantigens shared among patients and tumor types. Nat Biotechnol. (2024) 42:1107–17. doi:10.1038/s41587-023-01945-y. PMID: 37857725 PMC11251992

[18] Wang R Wu J Lin Y Xiao Y Yang B Yao S . An epitope-directed mRNA vaccine inhibits tumor metastasis through the blockade of MICA/B α1/2 shedding. Cell Rep Med. (2025) 6:101981. doi:10.1016/j.xcrm.2025.101981. PMID: 39999840 PMC11970329

[19] Chi H Carbone M Deng Y . When vaccines reset tumors: SARS-CoV-2 mRNA shots create a transient checkpoint-sensitive state. Sig Transduct Target Ther. (2025) 10:423. doi:10.1038/s41392-025-02521-3. PMID: 41469372 PMC12753749

[20] Zhang W Guan J Wang W Chen G Fan L Lu Z . Neoantigen-specific mRNA/DC vaccines for effective anticancer immunotherapy. Genes Immun. (2024) 25:514–24. doi:10.1038/s41435-024-00305-3. PMID: 39592852

[21] Zhang X Men K Zhang Y Zhang R Yang L Duan X . Local and systemic delivery of mRNA encoding survivin-T34A by lipoplex for efficient colon cancer gene therapy. Int J Nanomedicine. (2019) 14:2733–51. doi:10.2147/IJN.S198747. PMID: 31118608 PMC6503337

[22] Chiang C-L Benencia F Coukos G . Whole tumor antigen vaccines. Semin Immunol. (2010) 22:132–43. doi:10.1016/j.smim.2010.02.004. PMID: 20356763 PMC3119500

[23] Itoh T Ueda Y Kawashima I Nukaya I Fujiwara H Fuji N . Immunotherapy of solid cancer using dendritic cells pulsed with the HLA-A24-restricted peptide of carcinoembryonic antigen. Cancer Immunol Immunother. (2002) 51:99–106. doi:10.1007/s00262-001-0257-z. PMID: 11904734 PMC11032765

[24] Jain A Slansky JE Matey LC Allen HE Pardoll DM Schulick RD . Synergistic effect of a granulocyte-macrophage colony-stimulating factor–transduced tumor vaccine and systemic interleukin-2 in the treatment of murine colorectal cancer hepatic metastases. Ann Surg Oncol. (2003) 10:810–20. doi:10.1245/ASO.2003.10.006. PMID: 12900373

[25] Emens LA Asquith JM Leatherman JM Kobrin BJ Petrik S Laiko M . Timed sequential treatment with cyclophosphamide, doxorubicin, and an allogeneic granulocyte-macrophage colony-stimulating factor–secreting breast tumor vaccine: A chemotherapy dose-ranging factorial study of safety and immune activation. J Clin Oncol. (2009) 27:5911. doi:10.1200/JCO.2009.23.3494. PMID: 19805669 PMC2793039

[26] Lubaroff DM . Prostate cancer vaccines in clinical trials. Expert Rev Vaccines. (2012) 11:857–68. doi:10.1586/erv.12.54. PMID: 22913261

[27] Zheng L Edil BH Soares KC El-Shami K Uram JN Judkins C . A safety and feasibility study of an allogeneic colon cancer cell vaccine administered with a granulocyte–macrophage colony stimulating factor–producing bystander cell line in patients with metastatic colorectal cancer. Ann Surg Oncol. (2014) 21:3931–7. doi:10.1245/s10434-014-3844-x. PMID: 24943235 PMC4192092

[28] Oh J Barve M Matthews CM Koon EC Heffernan TP Fine B . Phase II study of Vigil® DNA engineered immunotherapy as maintenance in advanced stage ovarian cancer. Gynecol Oncol. (2016) 143:504–10. doi:10.1016/j.ygyno.2016.09.018. PMID: 27678295

[29] Foley CR Swan SL Swartz MA . Engineering challenges and opportunities in autologous cellular cancer immunotherapy. J Immunol. (2024) 212:188–98. doi:10.4049/jimmunol.2300642. PMID: 38166251 PMC11155266

[30] Banchereau J Steinman RM . Dendritic cells and the control of immunity. Nature. (1998) 392:245–52. doi:10.1038/32588. PMID: 9521319

[31] Radomski M Zeh HJ Edington HD Pingpank JF Butterfield LH Whiteside TL . Prolonged intralymphatic delivery of dendritic cells through implantable lymphatic ports in patients with advanced cancer. J Immunother Cancer. (2016) 4:24. doi:10.1186/s40425-016-0128-y. PMID: 27096100 PMC4835859

[32] Bernal-Estévez DA Ortíz Barbosa MA Ortíz-Montero P Cifuentes C Sánchez R Parra-López CA . Autologous dendritic cells in combination with chemotherapy restore responsiveness of T cells in breast cancer patients: A single-arm phase I/II trial. Front Immunol. (2021) 12:669965. doi:10.3389/fimmu.2021.669965. PMID: 34489928 PMC8417880

[33] Heras-Murillo I Mañanes D Munné P Núñez V Herrera J Catalá-Montoro M . Immunotherapy with conventional type-1 dendritic cells induces immune memory and limits tumor relapse. Nat Commun. (2025) 16:3369. doi:10.1038/s41467-025-58289-1. PMID: 40204706 PMC11982544

[34] Adamik J Munson PV Maurer DM Hartmann FJ Bendall SC Argüello RJ . Immuno-metabolic dendritic cell vaccine signatures associate with overall survival in vaccinated melanoma patients. Nat Commun. (2023) 14:7211. doi:10.1038/s41467-023-42881-4. PMID: 37938561 PMC10632482

[35] Lesterhuis WJ De Vries IJM Schreibelt G Schuurhuis DH Aarntzen EH De Boer A . Immunogenicity of dendritic cells pulsed with CEA peptide or transfected with CEA mRNA for vaccination of colorectal cancer patients. Anticancer Res. (2010) 30:5091–7. 21187495

[36] Bonehill A Van Nuffel AMT Corthals J Tuyaerts S Heirman C François V . Single-step antigen loading and activation of dendritic cells by mRNA electroporation for the purpose of therapeutic vaccination in melanoma patients. Clin Cancer Res. (2009) 15:3366–75. doi:10.1158/1078-0432.CCR-08-2982. PMID: 19417017

[37] Ma M Ac H T O D S D N Hk L . Depletion of human regulatory T cells specifically enhances antigen-specific immune responses to cancer vaccines. Blood. (2008) 112:610–618 . doi:10.1182/blood-2008-01-135319. PMID: 18519811 PMC2481547

[38] Har-Noy M Or R . Allo-priming as a universal anti-viral vaccine: protecting elderly from current COVID-19 and any future unknown viral outbreak. J Transl Med. (2020) 18:196. doi:10.1186/s12967-020-02363-3. PMID: 32398026 PMC7215129

[39] Hirschfeld A Gurell D Har-Noy M . Objective response after immune checkpoint inhibitors in a chemotherapy-refractory pMMR/MSS metastatic rectal cancer patient primed with experimental AlloStim® immunotherapy. Transl Med Commun. (2024) 9:15. doi:10.1186/s41231-024-00174-y. PMID: 38164791

[40] Fan T Zhang M Yang J Zhu Z Cao W Dong C . Therapeutic cancer vaccines: advancements, challenges and prospects. Signal Transduction Targeted Ther. (2023) 8:450. doi:10.1038/s41392-023-01674-3. PMID: 38086815 PMC10716479

[41] Romero P Banchereau J Bhardwaj N Cockett M Disis ML Dranoff G . The Human Vaccines Project: A roadmap for cancer vaccine development. Sci Transl Med. (2016) 8:334ps9–9. doi:10.1126/scitranslmed.aaf0685. PMID: 27075624

[42] Kaufman HL Kim DW Kim-Schulze S DeRaffele G Jagoda MC Broucek JR . Results of a randomized phase I gene therapy clinical trial of nononcolytic fowlpox viruses encoding T cell costimulatory molecules. Hum Gene Ther. (2014) 25:452. doi:10.1089/hum.2013.217. PMID: 24484178 PMC4027985

[43] Bilusic M Heery CR Arlen PM Rauckhorst M Apelian D Tsang KY . Phase I trial of a recombinant yeast-CEA vaccine (GI-6207) in adults with metastatic CEA-expressing carcinoma. Cancer Immunology Immunotherapy: CII. (2013) 63:225. doi:10.1007/s00262-013-1505-8. PMID: 24327292 PMC3944709

[44] Heery CR Singh BH Rauckhorst M Marté JL Donahue RN Grenga I . Phase I trial of a yeast-based therapeutic cancer vaccine (GI-6301) targeting the transcription factor brachyury. Cancer Immunol Res. (2015) 3:1248. doi:10.1158/2326-6066.CIR-15-0119. PMID: 26130065 PMC4636967

[45] Cohn A Morse MA O’Neil B Whiting S Coeshott C Ferraro J . Whole Recombinant Saccharomyces cerevisiae Yeast Expressing Ras Mutations as Treatment for Patients With Solid Tumors Bearing Ras Mutations: Results From a Phase 1 Trial. J Immunotherapy (Hagerstown Md: 1997). (2018) 41:141. doi:10.1097/CJI.0000000000000219. PMID: 29528991 PMC5895167

[46] Chen X Shi T Chen F Xie X Fang H Wu Z . Orally antigen-engineered yeast vaccine elicits robust intestinal mucosal immunity. ACS Nano. (2025) 19:10841–53. doi:10.1021/acsnano.4c14690. PMID: 40082064

[47] Nguyen KV Nguyen D-H Ngo H-T You S-H Kim S Hong Y . Salmonella typhimurium co-expressing cytolysin A and hyaluronidase suppresses tumor growth and metastasis. Cell Death Discov. (2026) 12:75. doi:10.1038/s41420-025-02897-9. PMID: 41484146 PMC12859083

[48] Nguyen D-H You S-H Ngo H-T Van Nguyen K Tran KV Chu T-H . Reprogramming the tumor immune microenvironment using engineered dual-drug loaded Salmonella. Nat Commun. (2024) 15:6680. doi:10.1038/s41467-024-50950-5. PMID: 39107284 PMC11303714

[49] Huang X Zhang G Tang T-Y Gao X Liang T-B . Personalized pancreatic cancer therapy: from the perspective of mRNA vaccine. Mil Med Res. (2022) 9:53. doi:10.1186/s40779-022-00416-w. PMID: 36224645 PMC9556149

[50] Viaud S Terme M Flament C Taieb J André F Novault S . Dendritic cell-derived exosomes promote natural killer cell activation and proliferation: a role for NKG2D ligands and IL-15Ralpha. PloS One. (2009) 4:e4942. doi:10.1371/journal.pone.0004942. PMID: 19319200 PMC2657211

[51] Campos-Mora M De Solminihac J Rojas C Padilla C Kurte M Pacheco R . Neuropilin-1 is present on Foxp3+ T regulatory cell-derived small extracellular vesicles and mediates immunity against skin transplantation. J Extracell Vesicles. (2022) 11:e12237. doi:10.1002/jev2.12237. PMID: 35676234 PMC9177693

[52] Poggio M Hu T Pai C-C Chu B Belair CD Chang A . Suppression of exosomal PD-L1 induces systemic anti-tumor immunity and memory. Cell. (2019) 177:414–427.e13. doi:10.1016/j.cell.2019.02.016. PMID: 30951669 PMC6499401

[53] Chen J Hu S Liu J Jiang H Wang S Yang Z . Exosomes: a double-edged sword in cancer immunotherapy. MedComm (2020). (2025) 6:e70095. doi:10.1002/mco2.70095. PMID: 39968497 PMC11831209

[54] A C J C H N M A Md M Ja H . Analysis of antigen presenting cell derived exosomes, based on immuno-magnetic isolation and flow cytometry. J Immunol Methods. (2001) 247:163–174 . doi:10.1016/s0022-1759(00)00321-5. PMID: 11150547

[55] Dai S Wei D Wu Z Zhou X Wei X Huang H . Phase I clinical trial of autologous ascites-derived exosomes combined with GM-CSF for colorectal cancer. Mol Ther. (2008) 16:782–90. doi:10.1038/mt.2008.1. PMID: 18362931 PMC7106337

[56] Meena SS Kosgei BK Soko GF Tingjun C Chambuso R Mwaiselage J . Developing anti-TDE vaccine for sensitizing cancer cells to treatment and metastasis control. NPJ Vaccines. (2025) 10:18. doi:10.1038/s41541-024-01035-3. PMID: 39870669 PMC11772600

[57] Turiello R Capone M Morretta E Monti MC Madonna G Azzaro R . Exosomal CD73 from serum of patients with melanoma suppresses lymphocyte functions and is associated with therapy resistance to anti-PD-1 agents. J Immunother Cancer. (2022) 10:e004043. doi:10.1136/jitc-2021-004043. PMID: 35273100 PMC8915288

[58] Lu T Zhang Z Zhang J Pan X Zhu X Wang X . CD73 in small extracellular vesicles derived from HNSCC defines tumour-associated immunosuppression mediated by macrophages in the microenvironment. J Extracell Vesicles. (2022) 11:e12218. doi:10.1002/jev2.12218. PMID: 35524455 PMC9077142

[59] Jeske R Liu C Duke L Canonicco Castro ML Muok L Arthur P . Upscaling human mesenchymal stromal cell production in a novel vertical-wheel bioreactor enhances extracellular vesicle secretion and cargo profile. Bioact Mater. (2023) 25:732–47. doi:10.1016/j.bioactmat.2022.07.004. PMID: 37056276 PMC10087597

[60] Zhang X Yuan X Shi H Wu L Qian H Xu W . Exosomes in cancer: small particle, big player. J Hematol Oncol. (2015) 8:83. doi:10.1186/s13045-015-0181-x. PMID: 26156517 PMC4496882

[61] Shah BA Holden JA Lenzo JC Hadjigol S O’Brien-Simpson NM . Multi-disciplinary approaches paving the way for clinically effective peptide vaccines for cancer. NPJ Vaccines. (2025) 10:68. doi:10.1038/s41541-025-01118-9. PMID: 40204832 PMC11982186

[62] Vansteenkiste JF Cho BC Vanakesa T De Pas T Zielinski M Kim MS . Efficacy of the MAGE-A3 cancer immunotherapeutic as adjuvant therapy in patients with resected MAGE-A3-positive non-small-cell lung cancer (MAGRIT): a randomised, double-blind, placebo-controlled, phase 3 trial. Lancet Oncol. (2016) 17:822–35. doi:10.1016/S1470-2045(16)00099-1. PMID: 27132212

[63] Hubbard JM Tőke ER Moretto R Graham RP Youssoufian H Lőrincz O . Safety and activity of PolyPEPI1018 combined with maintenance therapy in metastatic colorectal cancer: an open-label, multicenter, phase Ib study. Clin Cancer Res. (2022) 28:2818–29. doi:10.1158/1078-0432.CCR-22-0112. PMID: 35472243 PMC9365360

[64] Tsuruma T Hata F Torigoe T Furuhata T Idenoue S Kurotaki T . Phase I clinical study of anti-apoptosis protein, survivin-derived peptide vaccine therapy for patients with advanced or recurrent colorectal cancer. J Transl Med. (2004) 2:19. doi:10.1186/1479-5876-2-19. PMID: 15193151 PMC446218

[65] Kibe S Yutani S Motoyama S Nomura T Tanaka N Kawahara A . Phase II study of personalized peptide vaccination for previously treated advanced colorectal cancer. Cancer Immunol Res. (2014) 2:1154–62. doi:10.1158/2326-6066.CIR-14-0035. PMID: 25351849

[66] Malonis RJ Lai JR Vergnolle O . Peptide-based vaccines: current progress and future challenges. Chem Rev. (2020) 120:3210–29. doi:10.1021/acs.chemrev.9b00472. PMID: 31804810 PMC7094793

[67] Neeli P Maza PAMA Chai D Zhao D Hoi XP Chan KS . DNA vaccines against GPRC5D synergize with PD-1 blockade to treat multiple myeloma. NPJ Vaccines. (2024) 9:180. doi:10.1038/s41541-024-00979-w. PMID: 39353958 PMC11445568

[68] Xu L Ren W Wang Q Li J . Advances in nucleic acid universal influenza vaccines. Vaccines. (2024) 12:664. doi:10.3390/vaccines12060664. PMID: 38932393 PMC11209422

[69] Górecki DC Simons JP . The dangers of DNA vaccination. Nat Med. (1999) 5:126. doi:10.1038/5473. PMID: 9930843

[70] Li J Jiang R Wang J Wang X . Advances in mRNA vaccine therapy for breast cancer research. Discover Oncol. (2025) 16:673. doi:10.1007/s12672-025-02542-y. PMID: 40327249 PMC12055746

[71] Pardi N Hogan MJ Porter FW Weissman D . mRNA vaccines — a new era in vaccinology. Nat Rev Drug Discov. (2018) 17:261–79. doi:10.1038/nrd.2017.243. PMID: 29326426 PMC5906799

[72] Lundstrom K . Self-amplifying RNA viruses as RNA vaccines. Int J Mol Sci. (2020) 21:5130. doi:10.3390/ijms21145130. PMID: 32698494 PMC7404065

[73] Beissert T Perkovic M Vogel A Erbar S Walzer KC Hempel T . A trans-amplifying RNA vaccine strategy for induction of potent protective immunity. Mol Ther. (2020) 28:119–28. doi:10.1016/j.ymthe.2019.09.009. PMID: 31624015 PMC6953774

[74] Huang D Zhu X Ye S Zhang J Liao J Zhang N . Tumour circular RNAs elicit anti-tumour immunity by encoding cryptic peptides. Nature. (2024) 625:593–602. doi:10.1038/s41586-023-06834-7. PMID: 38093017

[75] Wang F Cai G Wang Y Zhuang Q Cai Z Li Y . Circular RNA-based neoantigen vaccine for hepatocellular carcinoma immunotherapy. MedComm. (2024) 5:e667. doi:10.1002/mco2.667. PMID: 39081513 PMC11286538

[76] Zhang M Zhao K Xu X Yang Y Yan S Wei P . A peptide encoded by circular form of LINC-PINT suppresses oncogenic transcriptional elongation in glioblastoma. Nat Commun. (2018) 9:4475. doi:10.1038/s41467-018-06862-2. PMID: 30367041 PMC6203777

[77] The Cancer Genome Atlas Network . Comprehensive molecular characterization of human colon and rectal cancer. Nature. (2012) 487:330–7. doi:10.1038/nature11252. PMID: 22810696 PMC3401966

[78] Li Y Wang M Peng X Yang Y Chen Q Liu J . mRNA vaccine in cancer therapy: current advance and future outlook. Clin Transl Med. (2023) 13:e1384. doi:10.1002/ctm2.1384. PMID: 37612832 PMC10447885

[79] Huang X Tang T Zhang G Liang T . Identification of tumor antigens and immune subtypes of cholangiocarcinoma for mRNA vaccine development. Mol Cancer. (2021) 20:50. doi:10.1186/s12943-021-01342-6. PMID: 33685460 PMC7938044

[80] Su X Fricke J Kavanagh D Irvine DJ . *In vitro* and *in vivo* mRNA delivery using lipid-enveloped pH-responsive polymer nanoparticles. Mol Pharmaceutics. (2011) 8:774. doi:10.1021/mp100390w. PMID: 21417235 PMC3354687

[81] Opitz AW Wickstrom E Thakur ML Wagner NJ . Physiologically based pharmacokinetics of molecular imaging nanoparticles for mRNA detection determined in tumor-bearing mice. Oligonucleotides. (2010) 20:117. doi:10.1089/oli.2009.0216. PMID: 20406142 PMC2966851

[82] Liao Z Wong SW Yeo HL Zhao Y . Smart nanocarriers for cancer treatment: clinical impact and safety. NanoImpact. (2020) 20:100253. doi:10.1016/j.impact.2020.100253. PMID: 38826717

[83] Bastin DJ Quizi J Kennedy MA Kekre N Auer RC . Current challenges in the manufacture of clinical-grade autologous whole cell vaccines for hematological Malignancies. Cytotherapy. (2022) 24:979–89. doi:10.1016/j.jcyt.2022.03.010. PMID: 35562303

[84] Kozak M Hu J . DNA vaccines: their formulations, engineering and delivery. Vaccines (Basel). (2024) 12:71. doi:10.3390/vaccines12010071. PMID: 38250884 PMC10820593

[85] Wang Y Zhang Z Luo J Han X Wei Y Wei X . mRNA vaccine: a potential therapeutic strategy. Mol Cancer. (2021) 20:33. doi:10.1186/s12943-021-01311-z. PMID: 33593376 PMC7884263

[86] Karikó K Buckstein M Ni H Weissman D . Suppression of RNA recognition by Toll-like receptors: the impact of nucleoside modification and the evolutionary origin of RNA. Immunity. (2005) 23:165–75. doi:10.1016/j.immuni.2005.06.008. PMID: 16111635

[87] Conry RM LoBuglio AF Wright M Sumerel L Pike MJ Johanning F . Characterization of a messenger RNA polynucleotide vaccine vector. Cancer Res. (1995) 55:1397–400. 7882341

[88] Kennel KB Greten FR . The immune microenvironment of colorectal cancer. Nat Rev Cancer. (2025) 25:945–64. doi:10.1038/s41568-025-00872-1. PMID: 40983666

[89] A C E L A B-L E E X H-M M I . Stromal gene expression defines poor-prognosis subtypes in colorectal cancer. Nat Genet. (2015) 47:320–329. doi:10.1038/ng.3225. PMID: 25706628

[90] A C E E S P-P Dv T M I Mv C . Dependency of colorectal cancer on a TGF-β-driven program in stromal cells for metastasis initiation. Cancer Cell. (2012) 22:571–584. doi:10.1016/j.ccr.2012.08.013. PMID: 23153532 PMC3512565

[91] Endo E Okayama H Saito K Nakajima S Yamada L Ujiie D . A TGFβ-dependent stromal subset underlies immune checkpoint inhibitor efficacy in DNA mismatch repair–deficient/microsatellite instability-high colorectal cancer. Mol Cancer Res. (2020) 18:1402–13. doi:10.1158/1541-7786.MCR-20-0308. PMID: 32493700

[92] Dvf T S P-P D S A B-L J B-R M I . TGFβ drives immune evasion in genetically reconstituted colon cancer metastasis. Nature. (2018) 554:538–543. doi:10.1038/nature25492. PMID: 29443964

[93] R J Sr H Jd L X C-L Jo L Ra R . Epithelial NOTCH signaling rewires the tumor microenvironment of colorectal cancer to drive poor-prognosis subtypes and metastasis. Cancer Cell. (2019) 36:319–336. doi:10.1016/j.ccell.2019.08.003. PMID: 31526760 PMC6853173

[94] Sampson N Brunner E Weber A Puhr M Schäfer G Szyndralewiez C . Inhibition of Nox4‐dependent ROS signaling attenuates prostate fibroblast activation and abrogates stromal‐mediated protumorigenic interactions. Int J Cancer. (2018) 143:383–95. doi:10.1002/ijc.31316. PMID: 29441570 PMC6067067

[95] Kc K S V Ch T Pc H . Metabolic communication in the tumour-immune microenvironment. Nat Cell Biol. (2022) 24:1574–1583. doi:10.1038/s41556-022-01002-x. PMID: 36229606

[96] Jh P Hj K Cw K Hc K Y J Hs L . Tumor hypoxia represses γδ T cell-mediated antitumor immunity against brain tumors. Nat Immunol. (2021) 22:336–346. doi:10.1038/s41590-020-00860-7. PMID: 33574616

[97] Vries N Haar J Veninga V Chalabi M Ijsselsteijn ME Ploeg M . γδ T cells are effectors of immunotherapy in cancers with HLA class I defects. Nature. (2023) 613:743. doi:10.1038/s41586-022-05593-1. PMID: 36631610 PMC9876799

[98] Tm B Lk Y E N Jm U X R Cj H . Tissue-specific reprogramming leads to angiogenic neutrophil specialization and tumor vascularization in colorectal cancer. J Clin Invest. (2024) 134:e174545. doi:10.1172/JCI174545. PMID: 38329810 PMC10977994

[99] J X J H J Z J P W L H Y . Lactylation-driven METTL3-mediated RNA m6A modification promotes immunosuppression of tumor-infiltrating myeloid cells. Mol Cell. (2022) 82:1660–1677. doi:10.1016/j.molcel.2022.02.033. PMID: 35320754

[100] Watson MJ Vignali PDA Mullett SJ Overacre-Delgoffe AE Peralta RM Grebinoski S . Metabolic support of tumour-infiltrating regulatory T cells by lactic acid. Nature. (2021) 591:645–52. doi:10.1038/s41586-020-03045-2. PMID: 33589820 PMC7990682

[101] Liang L Yang X Yao S Li X Wang F . Identification of lactylation-associated fibroblast subclusters predicting prognosis and cancer immunotherapy response in colon cancer. Gene. (2025) 940:149220. doi:10.1016/j.gene.2025.149220. PMID: 39765285

[102] Heil F Hemmi H Hochrein H Ampenberger F Kirschning C Akira S . Species-specific recognition of single-stranded RNA via toll-like receptor 7 and 8. Science. (2004) 303:1526–9. doi:10.1126/science.1093620. PMID: 14976262

[103] Yin Y Li X Ma H Zhang J Yu D Zhao R . In situ transforming RNA nanovaccines from polyethylenimine functionalized graphene oxide hydrogel for durable cancer immunotherapy. Nano Lett. (2021) 21:2224–31. doi:10.1021/acs.nanolett.0c05039. PMID: 33594887

[104] Scheel B Teufel R Probst J Carralot J-P Geginat J Radsak M . Toll-like receptor-dependent activation of several human blood cell types by protamine-condensed mRNA. Eur J Immunol. (2005) 35:1557–66. doi:10.1002/eji.200425656. PMID: 15832293

[105] Yoneyama M Kikuchi M Natsukawa T Shinobu N Imaizumi T Miyagishi M . The RNA helicase RIG-I has an essential function in double-stranded RNA-induced innate antiviral responses. Nat Immunol. (2004) 5:730–7. doi:10.1038/ni1087. PMID: 15208624

[106] Browne EP . Toll-like receptor 7 controls the anti-retroviral germinal center response. PloS Pathog. (2011) 7:e1002293. doi:10.1371/journal.ppat.1002293. PMID: 21998589 PMC3188541

[107] Miquel C-H Abbas F Cenac C Foret-Lucas C Guo C Ducatez M . B cell-intrinsic TLR7 signaling is required for neutralizing antibody responses to SARS-CoV-2 and pathogen-like COVID-19 vaccines. Eur J Immunol. (2023) 53:e2350437. doi:10.1002/eji.202350437. PMID: 37438976

[108] Wang T Song D Li X Luo Y Yang D Liu X . MiR-574-5p activates human TLR8 to promote autoimmune signaling and lupus. Cell Commun Signal. (2024) 22:220. doi:10.1186/s12964-024-01601-1. PMID: 38589923 PMC11000404

[109] Honey K . TLR ligands from the natural world. Nat Rev Immunol. (2004) 4:247. doi:10.1038/nri1337. PMID: 37880705

[110] Li Y Chen M Cao H Zhu Y Zheng J Zhou H . Extraordinary GU-rich single-strand RNA identified from SARS coronavirus contributes an excessive innate immune response. Microbes Infection. (2013) 15:88–95. doi:10.1016/j.micinf.2012.10.008. PMID: 23123977 PMC7110875

[111] Napolitani G Rinaldi A Bertoni F Sallusto F Lanzavecchia A . Selected Toll-like receptor agonist combinations synergistically trigger a T helper type 1–polarizing program in dendritic cells. Nat Immunol. (2005) 6:769–76. doi:10.1038/ni1223. PMID: 15995707 PMC3760217

[112] Clemens EA Holbrook BC McNeilly B Kanekiyo M Graham BS Alexander-Miller MA . TLR agonists induce sustained IgG to hemagglutinin stem and modulate T cells following newborn vaccination. NPJ Vaccines. (2022) 7:102. doi:10.1038/s41541-022-00523-8. PMID: 36038596 PMC9424286

[113] Hollingsworth RE Jansen K . Turning the corner on therapeutic cancer vaccines. NPJ Vaccines. (2019) 4:7. doi:10.1038/s41541-019-0103-y. PMID: 30774998 PMC6368616

[114] Mitchell DA Batich KA Gunn MD Huang M-N Sanchez-Perez L Nair SK . Tetanus toxoid and CCL3 improve DC vaccines in mice and glioblastoma patients. Nature. (2015) 519:366. doi:10.1038/nature14320. PMID: 25762141 PMC4510871

[115] He Q Gao H Tan D Zhang H Wang J . mRNA cancer vaccines: Advances, trends and challenges. Acta Pharm Sin B. (2022) 12:2969–89. doi:10.1016/j.apsb.2022.03.011. PMID: 35345451 PMC8942458

[116] Sahin U Oehm P Derhovanessian E Jabulowsky RA Vormehr M Gold M . An RNA vaccine drives immunity in checkpoint-inhibitor-treated melanoma. Nature. (2020) 585:107–12. doi:10.1038/s41586-020-2537-9. PMID: 32728218

[117] Van Lint S Goyvaerts C Maenhout S Goethals L Disy A Benteyn D . Preclinical evaluation of TriMix and antigen mRNA-based antitumor therapy. Cancer Res. (2012) 72:1661–71. doi:10.1158/0008-5472.CAN-11-2957. PMID: 22337996

[118] Wj L Ij de V Dh S Ac B Jf J Aj de B . Vaccination of colorectal cancer patients with CEA-loaded dendritic cells: antigen-specific T cell responses in DTH skin tests. Ann Oncol Off J Eur Soc For Med Oncol. (2006) 17:974–980. doi:10.1093/annonc/mdl072. PMID: 16600979

[119] L F Y H A R C B A Y Ga F . Altered peptide ligand vaccination with Flt3 ligand expanded dendritic cells for tumor immunotherapy. PNAS. (2001) 98:8809–8814. doi:10.1073/pnas.141226398. PMID: 11427731 PMC37517

[120] Tn S W S P K . Cancer neoantigens. Annu Rev Immunol. (2019) 37:173–200. doi:10.1146/annurev-immunol-042617-053402. PMID: 30550719

[121] Milo I Bedora-Faure M Garcia Z Thibaut R Périé L Shakhar G . The immune system profoundly restricts intratumor genetic heterogeneity. Sci Immunol. (2018) 3:eaat1435. doi:10.1126/sciimmunol.aat1435. PMID: 30470696

[122] Kim Y Park W Kim S Kim EH Choi J Jang H . Dual-targeting mRNA cancer vaccines for simultaneous antigen presentation in dendritic and tumor cells. ACS Nano. (2026) 20:9925–39. doi:10.1021/acsnano.5c20535. PMID: 41841891

[123] Canali S Fischer AW Nguyen M Anderson K Wu L Graham A-R . Lipid-encapsulated mRNA encoding an extended serum half-life interleukin-22 ameliorates metabolic disease in mice. Mol Metab. (2024) 86:101965. doi:10.1016/j.molmet.2024.101965. PMID: 38871178 PMC11296054

[124] Li N Li N Wang Y Cui N Wang X Tang Y . Preliminary safety, antitumor activity, and pharmacodynamics of intratumoral ABO2011 (IL-12 mRNA) in patients with advanced solid tumors. J Clin Oncol. (2024) 42:e14583–e14583. doi:10.1200/JCO.2024.42.16_suppl.e14583. PMID: 41909186

[125] Ashizawa N Takazono T Umeda M Yamamoto K Kawakami A Mukae H . Macrophage activation syndrome after BNT162b2 mRNA vaccination successfully treated with corticosteroids. Clin Exp Rheumatol. (2022) 40:1060. doi:10.55563/clinexprheumatol/a9hrmo. PMID: 35084316

[126] Lu S Zhang C Wu H Wang J Wang J Zhao L . A pH/MMP-9 smart dual-responsive liposome GBE@LP co-delivers and controls the release of GB1107/BMS1166/Enzalutamide for liver cancer immunotherapy. Mater Today Bio. (2025) 32:101801. doi:10.1016/j.mtbio.2025.101801. PMID: 40475856 PMC12139023

[127] X C J W B Z M Z J Z N Z . Bacterial lysate-based bifunctional mRNA nanoformulation for efficient colon cancer immunogene therapy. ACS Appl Materials Interfaces. (2024) 16:56580–56598. doi:10.1021/acsami.4c07684. PMID: 39397736

[128] Saxena M van der Burg SH Melief CJM Bhardwaj N . Therapeutic cancer vaccines. Nat Rev Cancer. (2021) 21:360–78. doi:10.1038/s41568-021-00346-0. PMID: 33907315

[129] Blankenstein T Coulie PG Gilboa E Jaffee EM . The determinants of tumour immunogenicity. Nat Rev Cancer. (2012) 12:307. doi:10.1038/nrc3246. PMID: 22378190 PMC3552609

[130] Goloudina A Le Chevalier F Authié P Charneau P Majlessi L . Shared neoantigens for cancer immunotherapy. Mol Ther Oncol. (2025) 33:200978. doi:10.1016/j.omton.2025.200978. PMID: 40256120 PMC12008704

[131] Fritah H Rovelli R Chiang C-L Kandalaft LE . The current clinical landscape of personalized cancer vaccines. Cancer Treat Rev. (2022) 106:102383. doi:10.1016/j.ctrv.2022.102383. PMID: 35367804

[132] Lopci E Hicks RJ Dimitrakopoulou-Strauss A Dercle L Iravani A Seban RD . Joint EANM/SNMMI/ANZSNM practice guidelines/procedure standards on recommended use of [18F]FDG PET/CT imaging during immunomodulatory treatments in patients with solid tumors version 1.0. Eur J Nucl Med Mol Imaging. (2022) 49:2323–41. doi:10.1007/s00259-022-05780-2. PMID: 35376991 PMC9165250

[133] Palmer CD Rappaport AR Davis MJ Hart MG Scallan CD Hong S-J . Individualized, heterologous chimpanzee adenovirus and self-amplifying mRNA neoantigen vaccine for advanced metastatic solid tumors: phase 1 trial interim results. Nat Med. (2022) 28:1619–29. doi:10.1038/s41591-022-01937-6. PMID: 35970920

[134] Mackensen A Haanen JB Koenecke C Alsdorf W Wagner-Drouet E Borchmann P . CLDN6-specific CAR-T cells plus amplifying RNA vaccine in relapsed or refractory solid tumors: the phase 1 BNT211–01 trial. Nat Med. (2023) 29:2844. doi:10.1038/s41591-023-02612-0. PMID: 37872225 PMC10667102

[135] New J Shenton L Ksayer R Wang J Zakharia K Nicholson LJ . Immune checkpoint inhibitors and vaccination: assessing safety, efficacy, and synergistic potential. Vaccines (Basel). (2024) 12:1270. doi:10.3390/vaccines12111270. PMID: 39591173 PMC11598700

[136] Widman AJ Cohen B Park V McClure T Wolchok J Kamboj M . Immune-related adverse events among COVID-19–vaccinated patients with cancer receiving immune checkpoint blockade. J Natl Compr Cancer Network. (2022) 20:1134–8. doi:10.6004/jnccn.2022.7048. PMID: 36240845 PMC10975484

[137] Kocher K Drost F Tesfaye AM Moosmann C Schülein C Grotz M . Vaccination-induced T cell responses maintain polyclonality with high antigen receptor avidity. Sci Immunol. (2025) 10(112):eadu6730. doi:10.1126/sciimmunol.adu6730. PMID: 41042909

[138] Hecht JR Spira AI Nguyen AV Berim LD Starodub A Pelster M . A randomized phase 2 study of an individualized neoantigen-targeting immunotherapy in patients with newly diagnosed metastatic microsatellite stable colorectal cancer (MSS-CRC). J Clin Oncol. (2025) 43:LBA13–LBA13. doi:10.1200/JCO.2025.43.4_suppl.LBA13. PMID: 41909186

[139] Ascierto PA Melero I . Reframing adjuvant immunotherapy in melanoma: all of it starts with priming. J Immunother Cancer. (2025) 13:e013766. doi:10.1136/jitc-2025-013766. PMID: 41365535 PMC12699564

[140] Grippin AJ Marconi C Copling S Li N Braun C Woody C . SARS-CoV-2 mRNA vaccines sensitize tumours to immune checkpoint blockade. Nature. (2025) 647:488–97. doi:10.1038/s41586-025-09655-y. PMID: 41125896 PMC12611756

[141] Wesselhoeft RA Kowalski PS Parker-Hale FC Huang Y Bisaria N Anderson DG . RNA circularization diminishes immunogenicity and can extend translation duration *in vivo*. Mol Cell. (2019) 74:508–520.e4. doi:10.1016/j.molcel.2019.02.015. PMID: 30902547 PMC6724735

[142] Chen YG Chen R Ahmad S Verma R Kasturi SP Amaya L . N6-methyladenosine modification controls circular RNA immunity. Mol Cell. (2019) 76:96–109.e9. doi:10.1016/j.molcel.2019.07.016. PMID: 31474572 PMC6778039

[143] Liu C-X Guo S-K Nan F Xu Y-F Yang L Chen L-L . RNA circles with minimized immunogenicity as potent PKR inhibitors. Mol Cell. (2022) 82:420–434.e6. doi:10.1016/j.molcel.2021.11.019. PMID: 34951963

[144] Gong Z Hu W Zhou C Guo J Yang L Wang B . Recent advances and perspectives on the development of circular RNA cancer vaccines. NPJ Vaccines. (2025) 10:41. doi:10.1038/s41541-025-01097-x. PMID: 40025038 PMC11873252

[145] Zhang Q Luo J Wu S Si H Gao C Xu W . Prognostic and predictive impact of circulating tumor DNA in patients with advanced cancers treated with immune checkpoint blockade. Cancer Discov. (2020) 10:1842–53. doi:10.1158/2159-8290.CD-20-0047. PMID: 32816849 PMC8358981

[146] Boland CR Goel A . Microsatellite instability in colorectal cancer. Gastroenterology. (2010) 138:2073–2087.e3. doi:10.1053/j.gastro.2009.12.064. PMID: 20420947 PMC3037515

[147] Salem ME Bodor JN Puccini A Xiu J Goldberg RM Grothey A . Relationship between MLH1, PMS2, MSH2 and MSH6 gene-specific alterations and tumor mutational burden in 1057 microsatellite instability-high solid tumors. Int J Cancer. (2020) 147:2948–56. doi:10.1002/ijc.33115. PMID: 32449172 PMC7530095

[148] Palles C Cazier J-B Howarth KM Domingo E Jones AM Broderick P . Germline mutations in the proof-reading domains of POLE and POLD1 predispose to colorectal adenomas and carcinomas. Nat Genet. (2013) 45:136–44. doi:10.1038/ng.2503. PMID: 23263490 PMC3785128

[149] Tan H Yu T Liu C Wang Y Jing F Ding Z . Identifying tumor antigens and immuno‐subtyping in colon adenocarcinoma to facilitate the development of mRNA vaccine. Cancer Med. (2022) 11:4656–72. doi:10.1002/cam4.4846. PMID: 35593226 PMC9741973

[150] Blass E Ott PA . Advances in the development of personalized neoantigen-based therapeutic cancer vaccines. Nat Rev Clin Oncol. (2021) 18:215–29. doi:10.1038/s41571-020-00460-2. PMID: 33473220 PMC7816749

[151] McGranahan N Furness AJS Rosenthal R Ramskov S Lyngaa R Saini SK . Clonal neoantigens elicit T cell immunoreactivity and sensitivity to immune checkpoint blockade. Science. (2016) 351:1463–9. doi:10.1126/science.aaf1490. PMID: 26940869 PMC4984254

[152] Stanley J Lakatos E Baker A-M Cross W Hart A Graham T . O15 evolutionary characteristics of neoantigens in inflammatory bowel disease and colorectal cancer. Gut. (2021) 70:A8–9. doi:10.1136/gutjnl-2020-bsgcampus.15

[153] Galbraith AJ Titmuss E Topham JT Tu D Renouf DJ Schaeffer DF . The interplay of HLA diversity and copy loss, T-cell profiles, and immunotherapy efficacy in colorectal adenocarcinoma. J Clin Oncol. (2026) 44:196. doi:10.1200/JCO.2026.44.2_suppl.196

[154] McGranahan N Rosenthal R Hiley CT Rowan AJ Watkins TBK Wilson GA . Allele-specific HLA loss and immune escape in lung cancer evolution. Cell. (2017) 171:1259–1271.e11. doi:10.1016/j.cell.2017.10.001. PMID: 29107330 PMC5720478

[155] Guinney J Dienstmann R Wang X de Reyniès A Schlicker A Soneson C . The consensus molecular subtypes of colorectal cancer. Nat Med. (2015) 21:1350–6. doi:10.1038/nm.3967. PMID: 26457759 PMC4636487

[156] Yamazaki T Gunderson AJ Gilchrist M Whiteford M Kiely MX Hayman A . Galunisertib plus neoadjuvant chemoradiotherapy in patients with locally advanced rectal cancer: a single-arm, phase 2 trial. Lancet Oncol. (2022) 23:1189–200. doi:10.1016/S1470-2045(22)00446-6. PMID: 35952709

[157] Yao S Han Y Yang M Jin K Lan H . It’s high-time to re-evaluate the value of induced-chemotherapy for reinforcing immunotherapy in colorectal cancer. Front Immunol. (2023) 14:1241208. doi:10.3389/fimmu.2023.1241208. PMID: 37920463 PMC10619163

[158] Nicolas AM Pesic M Engel E Ziegler PK Diefenhardt M Kennel KB . Inflammatory fibroblasts mediate resistance to neoadjuvant therapy in rectal cancer. Cancer Cell. (2022) 40:168–184.e13. doi:10.1016/j.ccell.2022.01.004. PMID: 35120600

[159] Bencsikova B Budinska E Selingerova I Pilatova K Fedorova L Greplova K . Circulating T cell subsets are associated with clinical outcome of anti-VEGF-based 1st-line treatment of metastatic colorectal cancer patients: a prospective study with focus on primary tumor sidedness. BMC Cancer. (2019) 19:687. doi:10.1186/s12885-019-5909-5. PMID: 31307428 PMC6631500

[160] Miao L Li L Huang Y Delcassian D Chahal J Han J . Delivery of mRNA vaccines with heterocyclic lipids increases anti-tumor efficacy by STING-mediated immune cell activation. Nat Biotechnol. (2019) 37:1174–85. doi:10.1038/s41587-019-0247-3. PMID: 31570898

[161] Choi H Lee S Kim H Bae S-H Jo S Kim J . Integration of TLR7/8 agonists into lipid nanoparticles enhances antigen-specific immune responses to N1-methyl-Ψ-modified mRNA-LNP vaccines. J Biol Eng. (2025) 19:103. doi:10.1186/s13036-025-00573-1. PMID: 41257870 PMC12629014

[162] Baiersdörfer M Boros G Muramatsu H Mahiny A Vlatkovic I Sahin U . A facile method for the removal of dsRNA contaminant from *in vitro*-transcribed mRNA. Mol Ther Nucleic Acids. (2019) 15:26–35. doi:10.1016/j.omtn.2019.02.018. PMID: 30933724 PMC6444222

[163] Karikó K Muramatsu H Ludwig J Weissman D . Generating the optimal mRNA for therapy: HPLC purification eliminates immune activation and improves translation of nucleoside-modified, protein-encoding mRNA. Nucleic Acids Res. (2011) 39:e142. doi:10.1093/nar/gkr695. PMID: 21890902 PMC3241667

[164] Hou X Zaks T Langer R Dong Y . Lipid nanoparticles for mRNA delivery. Nat Rev Mater. (2021) 6:1078–94. doi:10.1038/s41578-021-00358-0. PMID: 34394960 PMC8353930

[165] Gulati GK Simpson AC MacMillen Z Krieger K Sharma S Erasmus JH . Preclinical development of lyophilized self-replicating RNA vaccines for COVID-19 and malaria with improved long-term thermostability. J Control Release. (2025) 377:81–92. doi:10.1016/j.jconrel.2024.11.023. PMID: 39547422 PMC11663110

[166] Stewart-Jones GBE Elbashir SM Wu K Lee D Renzi I Ying B . Domain-based mRNA vaccines encoding spike protein N-terminal and receptor binding domains confer protection against SARS-CoV-2. Sci Transl Med. (2023) 15:eadf4100. doi:10.1126/scitranslmed.adf4100. PMID: 37703353

[167] Kübler H Scheel B Gnad-Vogt U Miller K Schultze-Seemann W Vom Dorp F . Self-adjuvanted mRNA vaccination in advanced prostate cancer patients: a first-in-man phase I/IIa study. J Immunother Cancer. (2015) 3:26. doi:10.1186/s40425-015-0068-y. PMID: 26082837 PMC4468959

[168] Miao L Zhang Y Huang L . mRNA vaccine for cancer immunotherapy. Mol Cancer. (2021) 20:41. doi:10.1186/s12943-021-01335-5. PMID: 33632261 PMC7905014

[169] Wang B Pei J Xu S Liu J Yu J . Recent advances in mRNA cancer vaccines: meeting challenges and embracing opportunities. Front Immunol. (2023) 14:1246682. doi:10.3389/fimmu.2023.1246682. PMID: 37744371 PMC10511650

